# A New Calmodulin-Binding Protein Expresses in the Context of Secondary Cell Wall Biosynthesis and Impacts Biomass Properties in *Populus*

**DOI:** 10.3389/fpls.2018.01669

**Published:** 2018-12-05

**Authors:** Raghuram Badmi, Raja S. Payyavula, Garima Bali, Hao-Bo Guo, Sara S. Jawdy, Lee E. Gunter, Xiaohan Yang, Kimberly A. Winkeler, Cassandra Collins, William H. Rottmann, Kelsey Yee, Miguel Rodriguez, Robert W. Sykes, Stephen R. Decker, Mark F. Davis, Arthur J. Ragauskas, Gerald A. Tuskan, Udaya C. Kalluri

**Affiliations:** ^1^BioEnergy Science Center, Oak Ridge National Laboratory, Oak Ridge, TN, United States; ^2^The Center for Bioenergy Innovation and Biosciences Division, Oak Ridge National Laboratory, Oak Ridge, TN, United States; ^3^Georgia Institute of Technology, Atlanta, GA, United States; ^4^Department of Biochemistry and Cellular and Molecular Biology, The University of Tennessee, Knoxville, Knoxville, TN, United States; ^5^ArborGen Inc., Ridgeville, SC, United States; ^6^National Renewable Energy Laboratory, Golden, CO, United States; ^7^Department of Chemical and Biomolecular Engineering, The University of Tennessee, Knoxville, Knoxville, TN, United States

**Keywords:** secondary cell wall, cellulose, signaling, kinesin, calcium-calmodulin, biomass, IQD, *Populus*

## Abstract

A greater understanding of biosynthesis, signaling and regulatory pathways involved in determining stem growth and secondary cell wall chemistry is important for enabling pathway engineering and genetic optimization of biomass properties. The present study describes a new functional role of *PdIQD10*, a *Populus* gene belonging to the IQ67-Domain1 family of *IQD* genes, in impacting biomass formation and chemistry. Expression studies showed that *PdIQD10* has enhanced expression in developing xylem and tension-stressed tissues in *Populus deltoides*. Molecular dynamics simulation and yeast two-hybrid interaction experiments suggest interactions with two calmodulin proteins, CaM247 and CaM014, supporting the sequence-predicted functional role of the PdIQD10 as a calmodulin-binding protein. PdIQD10 was found to interact with specific *Populus* isoforms of the Kinesin Light Chain protein family, shown previously to function as microtubule-guided, cargo binding and delivery proteins in *Arabidopsis*. Subcellular localization studies showed that PdIQD10 localizes in the nucleus and plasma membrane regions. Promoter-binding assays suggest that a known master transcriptional regulator of secondary cell wall biosynthesis (PdWND1B) may be upstream of an *HD-ZIP III* gene that is in turn upstream of *PdIQD10* gene in the transcriptional network. RNAi-mediated downregulation of *PdIQD10* expression resulted in plants with altered biomass properties including higher cellulose, wall glucose content and greater biomass quantity. These results present evidence in support of a new functional role for an *IQD* gene family member, *PdIQD10*, in secondary cell wall biosynthesis and biomass formation in *Populus.*

## Introduction

Plant cell walls play essential structural and functional roles as strength-conferring and signal-responsive organelles made up of polysaccharides (cellulose, hemicellulose and pectin); polyphenols; lignin and polypeptides (wall glycoproteins and wall associated proteins). A greater understanding of the biosynthesis, signaling and regulatory pathways involved in the coordinated deposition and remodeling of plant cell walls is of great interest to fundamental plant science as well as applied biomass improvement research fields (Demura and Ye, 2010; Mizrachi et al., 2012). Improvements in the precision of pathway engineering efforts and acceleration in breeding approaches will need complementation of the existing knowledgebase of known cell wall pathway genes with functional characterization of genes that co-express with known marker cell wall pathway genes (Ruprecht et al., 2011; Sibout et al., 2017).

The primary characteristics that render a given unit of lignocellulosic biomass more amenable to conversion to biofuel include higher content and greater accessibility to the wall glucose or cellulose, reduced lignin content and cross-linkages among cellulose, lignin and hemicellulose (Klemm et al., 2005; McCann and Carpita, 2008; Mansfield, 2009; Scheller and Ulvskov, 2010; Fu et al., 2011; Ding et al., 2012; Cragg et al., 2015; Busse-Wicher et al., 2016; Gall et al., 2017). Toward the goal of identifying high potential candidate genes involved in signaling and regulation of biosynthesis of cell walls with high substrate content, we studied overlaps in expression data collected from developmental (xylem development) and physiological response (tension stress) phases when more wood cells, thicker cell walls and walls with higher cellulose content are formed. These include three studies; firstly, a tension stress response profiling study to characterize transcriptome response to bending/leaning in stems of *Populus*, while undergoing enhanced xylem cell proliferation and cellulose production in new cell wall layers composed of over 90% cellulose (Abraham et al., 2012). Secondly, a xylem proteomics study to profile a tissue type where enhanced cellulose and secondary cell wall production occurs (Kalluri et al., 2009). Lastly, a co-expression network analysis study to identify tightly co-expressed genes with a *Populus* secondary *CesA* gene (Yang et al., 2011). *PdIQD10* (Potri.001G375700) gene, predicted to code for a calmodulin-binding protein, was identified as highly upregulated during phases of enhanced cellulose biosynthesis.

Calcium (Ca^2+^)-related signaling pathways constitute a major cellular signaling mechanism in response to a stress or developmental trigger and are prevalent among all eukaryotes (Clapham, 2007; Dodd et al., 2010). Ca^2+^ ion levels serve as important secondary messengers by inducing intracellular dose-dependent signals that are transduced or decoded via Ca^2+^ sensor proteins. Ca^2+^-dependent protein kinases (CDPKs), CaMs and CMLs, and CBLs are the three major Ca^2+^ sensor proteins in plants. CDPKs have an intrinsic kinase domain that can directly transduce the signal to the target proteins upon sensing the Ca^2+^ signal; whereas, CaMs/CMLs and CBLs trigger a conformational change in their structure upon Ca^2+^ perception and interact with their target proteins to transduce the Ca^2+^ signal. CBLs interact specifically with CIPKs to transduce the Ca^2+^ signals for various intracellular processes or responses. CaMs are a large class of Ca^2+^ sensor proteins with 7 *CaM* and 50 *CML* genes encoded in the *Arabidopsis* genome (Abel et al., 2013; Bürstenbinder et al., 2013). CaMs and CMLs are known to interact with wide array of proteins with varied functions such as metabolic enzymes, transcriptional regulators, protein kinases, cytoskeletal proteins and ion transporters (Snedden and Fromm, 2001). In *Populus*, members of the Ca(^2+^)-calmodulin module are known to play important roles in induction of freeze tolerance (Lin et al., 2004) and salt stress (Chang et al., 2006) responses.

Recent studies have described a new plant-specific class of calmodulin interacting proteins with conserved IQ-67 domains, referenced after the isoleucine and glutamine (IQ)- amino acid rich region and the central domain of 67 conserved amino acid residues (Abel et al., 2005; Levy et al., 2005; Bürstenbinder et al., 2013). These conserved IQD-67 domain containing proteins are referred to as IQDs and belong to a structurally conserved large gene family of 33 predicted members in *Arabidopsis* (Abel et al., 2005). Although, previously identified calmodulin-binding proteins were Ca^2+^-dependent, further studies revealed the occurrence of Ca^2+^-independent calmodulin interacting proteins. One such example is of intestinal brush border myosin I, which interacts with Ca^2+^-free form of calmodulin called ‘apocalmodulin’ (Bahler and Rhoads, 2002). Members of IQD family also appear to interact with CaMs in Ca^2+^-dependent and Ca^2+^-independent manner (Bahler and Rhoads, 2002; Abel et al., 2005). The genome of *Populus trichocarpa* encodes for 40 *IQD* genes (Hui et al., 2014).

Co-expression studies in *Arabidopsis* have previously reported the sequence ortholog of *PdIQD10*, *AtIQD10* (AT3G15050), as co-expressed with *AtCesA*s as well as *AtSND1*, *AtNST1*, and *AtMYB103*, a set of transcription factors involved in secondary cell wall biosynthesis (Endler and Persson, 2011; Xu et al., 2013). However, no functional evidence for involvement in either biosynthesis or signaling pathways related to secondary cell wall formation have been found. While these co-expression studies suggest a potential role of an *IQD* gene in the context of cell wall biosynthesis, functional evidence at molecular, cellular or plant level has not been reported.

Functional evidence for a distinct *Arabidopsis IQD* gene family member, *AtIQD1*, has been presented in the context of glucosinolate metabolism and defense response as well as cell growth and microtubule organization (Levy et al., 2005; Abel et al., 2013; Bürstenbinder et al., 2013, 2017b). AtIQD1 was shown to interact with KLCR1 or KLCs and localize to microtubules as well as to nucleus (Bürstenbinder et al., 2013). It is proposed that the cargo transport function of kinesin is activated over microtubule cross-link or sliding function in the presence of KLCs (Wong and Rice, 2010). AtIQD1 was hypothesized to function as a molecular scaffold in cargo transport across microtubule tracks of the cell (Hirokawa et al., 2009; Verhey et al., 2011; Bürstenbinder et al., 2013). The current understanding of the cellular and plant functional context of IQD proteins is limited and primarily derived from a research study by Bürstenbinder et al. (2013). Considering that the Ca^2+^/calmodulin signaling system is integral in mediating a diverse range of plant processes (Bürstenbinder et al., 2017a), individual members of the large *IQD* gene family may be differentiated to express and function in the context of distinct developmental and physiological phases.

Here, we present evidence in support of a new functional role for an *IQD* gene family member, *PdIQD10*, in secondary cell wall biosynthesis and biomass formation in *Populus deltoides*.

## Materials and Methods

### Phylogenetic and Sequence Analysis

Protein sequences of *Populus* IQD and CaM (Table 1) homologs were retrieved from *Phytozome v9.1*: *Populus trichocarpa v3.0*, and the NCBI database. Phylogenetic analysis was performed in the MEGA 7.0.25 (Molecular Evolutionary Genetics Analysis) program using the Maximum Likelihood method (Payyavula et al., 2014; Kumar et al., 2016). Bootstrap values were calculated from 1000 independent bootstrap runs. Protein sequence alignment was performed using Clustal W.

**Table 1:** List of calmodulin and calmodulin-like proteins employed in the study.

Sl. No	*Populus* Potri ID	Gene names in this paper	Cloned and tested	*Arabidopsis* orthologs
1	Potri.016G024700.2	CaM247	Yes	AT3G43810.1
2	Potri.001G117900.1	CaM1179	Yes	AT1G12310.1
3	Potri.T124900.1	CaM1249	Yes	AT3G07490.1
4	Potri.004G035100.1	CaM351	Yes	AT5G57580
5	Potri.005G128100.1	CaM1281	Yes	AT3G50770.1
6	Potri.008G134300.1	CaM1343	Yes	AT1G24620.1
7	Potri.017G029700.1	CaM297	Yes	AT3G07490.1
8	Potri.014G093900.1	CaM939	Yes	AT4G01150.1
9	Potri.007G031900.1	CaM319	Yes	AT3G50770.1
10	Potri.006G043700.1	CaM437	Yes	AT3G10190.1
11	Potri.004G122900.1	CaM1229	Yes	AT5G39670.1
12	Potri.001G314900.1	CaM3149	Yes	AT3G24110.1
13	Potri.013G040300.1	CaM403	Yes	AT3G22930.1
14	Potri.002G047300.1	CaM473	Yes	AT3G22930.1
15	Potri.002G239100.1	CaM2391	Yes	AT3G07490.1
16	Potri.015G032600.1	CaM326	Yes	AT5G37780.1
17	Potri.005G052800.1	CaM528	Yes	AT4G14640.1
18	Potri.012G041000.1	CaM410	Yes	AT5G37780.1
19	Potri.009G021500.1	CaM215	Yes	AT2G27030.3
20	Potri.003G095700.1	CaM957	Yes	AT1G32250.1
21	Potri.015G052600.1	CaM526	Yes	AT1G18530.1
22	Potri.002G001400.1	CaM014	Yes	
23	Potri.006G065900.1	CaM659	Yes	
24	Potri.005G259900.1	CaM2599	Yes	AT5G42380.1
25	Potri.012G048200.1	CaM482	Yes	AT1G18210.1
26	Potri.006G026700.1	CaM267	Yes	
27	Potri.006G043900.1	CaM439	No	AT5G04020.1
28	Potri.016G040900.1	CaM409	No	AT3G10190.1
29	Potri.001G375400.1	CaM3754	No	AT1G53210.1
30	Potri.017G054900.1	CaM549	No	AT3G24110.1
31	Potri.001G332900.1	CaM3329	No	AT1G76650.1
32	Potri.002G239200.1	CaM2392	No	AT3G25440.1
33	Potri.007G128600.1	CaM1286	No	AT2G43290.1
34	Potri.001G222200.1	CaM2222	No	AT3G56800.1
35	Potri.003G116800.1	CaM1168	No	AT1G24620.1
36	Potri.007G042700.1	CaM427	No	


### Construct Development and Plant Transformation

The *PdIQD10* RNAi construct was developed by PCR-amplifying a 156 bp nucleotide sequence overlapping the 3′ coding and UTR regions (Supplementary Figure S1), cloning into a Gateway entry vector and then binary vector via LR Clonase recombination were transformed into wild-type *P. deltoides* ‘WV94’ according to previously published methods for *Agrobacterium*-based transformation of *Populus* (Meilan and Ma, 2006; Kumar et al., 2016). For subcellular localization, the full length protein-coding sequence corresponding to *PdIQD10* (Potri.001G375700) was amplified from the *P. deltoides* xylem cDNA library (primers are listed in Table 2) using Q5 High-Fidelity DNA polymerase (New England Biolabs, Ipswich, MA, United States), and cloned in a pENTR vector (Invitrogen, Carlsbad, CA, United States). After sequence confirmation, the coding region fragments were recombined into one of the Gateway binary vectors pGWB405, pGWB444 and pGWB454 (Waadt and Kudla, 2008; Nakagawa et al., 2009) using LR clonase (Invitrogen), and the plasmid DNA from a single colony each was used to transform *Agrobacterium*. Tobacco infiltration and protein localization were performed as described previously (Waadt and Kudla, 2008). *Agrobacterium* harboring binary constructs of *PdIQD10*, *CaM014* and *CaM247* were cultured overnight in LB media. After a brief centrifugation, the supernatant was removed and the pellet was dissolved in 10 mM MgCl_2_ and OD at A_600_ was adjusted to 0.5. The culture was infiltrated into 4-week old tobacco leaves. After 48–72 h, roughly 4 mm^2^ leaf sections were cut and fixed in 3.7% formaldehyde, 50 mM NaH_2_PO_4_ and 0.2% Triton X-100 for 30 min, then rinsed with phosphate-buffered saline (PBS) and stained in DAPI (4,6′-diamidino-2-phenylindole, 1.5 μg ml^-1^ in PBS) for 30 min. For protoplast transfection experiments, CaM247 and CaM014 were cloned into CD3-1654 vector obtained from the Arabidopsis Biological Resource Center. *Populus* protoplasts were isolated and transfected as described in Guo et al. (2012). FM-64 (#T13320) was used as plasma membrane marker and mCherry-VirD2NLS was used as nuclear marker. Fluorescence visualization and imaging was performed on a Zeiss LSM710 confocal laser scanning microscope (Carl Zeiss Microscopy, Thornwood, NY, United States) equipped with a Plan-Apochromat 63x/1.40 oil immersion objective. To increase the accessibility of images obtained from these subcellular localization experiments (Wong, 2011), the yellow color channel was converted to magenta uniformly across all images in the CMYK color spectrum. The original RGB color scheme images are also provided in Supplemental Files.

**Table 2:** List of primers used in the study.

Sl No.	Primer name	Primer sequence (5′-3′)	Purpose
1	IQD10-RNAiF	CACCCCCGGGGCTGTGGATTGTCAAGCTG	RNAi construct
2	IQD10-RNAiR	TCTAGATTTACACGCTGCATGCCA	RNAi construct
3	IQD10-IF-BDF	CATGGAGGCCGAATTCATGGGTTGTCTCTTCTCTGGG	Yeast two-hybrid
4	IQD10-IF-BDR	GCAGGTCGACGGATCCACTTGGAATCAGCAGATGAGAC	
5	KLC400-IF-ADF	GGAGGCCAGTGAATTCATGTTGGGCCTAGTCTCTGC	
6	KLC400-IF-ADR	TCATCTGCAGCTCGAGCGAGCTAGAATCTAGGAGATTTAC	
7	KLC3200-IF-ADF	GGAGGCCAGTGAATTCATGCCAGGTCTTGTCTCTG	
8	KLC3200-IF-ADR	TCATCTGCAGCTCGAGAATTCTAAAACCCCACTTC	
9	KLC4700-ADF	GGAGGCCAGTGAATTCATGCCGGGACTAGCCATGG	
10	KLC4700-ADR	TCATCTGCAGCTCGAGCGAGACCTCAAGATCATCG	
11	KLC7800-ADF	GGAGGCCAGTGAATTCATGCCTGGTCTTGTCTCTG	
12	KLC7800-ADR	TCATCTGCAGCTCGAGAATTCTAAAACCCCACTTC	
13	MAP599-ADF	GGAGGCCAGTGAATTCATGGAGAAAGCACATACAAAATC	
14	MAP599-ADR	TCATCTGCAGCTCGAGCTTAACAGGCTTCCAGGCATG	
15	DICE1-ADF	GGAGGCCAGTGAATTCATGCTGTACCGACGGGACCTG	
16	DICE1-ADR	TCATCTGCAGCTCGAGCCTGTCTAACTTTACATGTGC	
17	Chi1416-ADF	GGAGGCCAGTGAATTCATGGAGGCCAAATGGTGTTTTC	
18	Chi1416-ADR	TCATCTGCAGCTCGAGAGAAGATGATGCAGAGGCAGAG	
21	CHUP1-ADF	GGAGGCCAGTGAATTCATGCGATTCCAATCTCCTG	
22	CHUP1-ADR	TCATCTGCAGCTCGAGTTGTTTGAGACTACCCATGC	
23	RIC4-ADF	GGAGGCCAGTGAATTCATGCATGCATGCCCCTTTCAG	
24	RIC4-ADR	TCATCTGCAGCTCGAGAGAGAGCCTGGAATGATCGACC	
25	HYP2316-ADF	GGAGGCCAGTGAATTCATGGGAAGTGAGCGGTGCAATA	
26	HYP2316-ADR	TCATCTGCAGCTCGAGCATGGGCAACCGGAGAAGTGAC	
27	UC2-ADF	GGAGGCCAGTGAATTCATGGCCGGTTTAATTTCAAGATC	
28	UC2-ADR	TCATCTGCAGCTCGAGGAAAACCATCACAAAAACGGCG	
29	Lac17-ADF	GGAGGCCAGTGAATTCATGGGTGCTTCATTTCTTCCATC	
30	Lac17-ADR	TCATCTGCAGCTCGAGACACTTGGGAAGATCGGCTGG	
31	PtiCesA7-A-ADF	GGAGGCCAGTGAATTCATGGAAGCCAGTGCTGGACTTG	
32	PtiCesA7-A-ADR	TCATCTGCAGCTCGAGACAGTTGATTCCACATTGCTTG	
33	MAP1733-ADF	GGAGGCCAGTGAATTCATGGGTTCGTTCCAAATTGG	
34	MAP1733-ADR	TCATCTGCAGCTCGAGGGGAGATAACGGCCCAGAAAAGG	
35	PdGAUT12.1-ADF	GGAGGCCAGTGAATTCATGCAGCTTCATATATCGCCGAG	
36	PdGAUT12.1-ADR	TCATCTGCAGCTCGAGAGATGGCCTAATATGACAGCC	
37	IRX10-ADF	GGAGGCCAGTGAATTCATGAGGACATGCTTGTGGG	
38	IRX10-ADR	TCATCTGCAGCTCGAGCCAAGGTTTCAGGTCCCCAACTG	
39	NEK5-ADF	GGAGGCCAGTGAATTCATGGAGGCAGATACCGGTGAAG	
40	NEK5-ADR	TCATCTGCAGCTCGAGGGTCCCTCCATTAATTTTTTTTTTAGAC	
41	NEK6-ADF	GGAGGCCAGTGAATTCATGGAGGCAGATGCTGCTGAAG	
42	NEK6-ADR	TCATCTGCAGCTCGAGGGTCCCTTCATTGAGTTTTTGC	
43	PdSCPL14-ADF	GGAGGCCAGTGAATTCATGGATTTGGTACCCAAGGTG	
44	PdSCPL14-ADR	TCATCTGCAGCTCGAGTCGTTCACAAGGAAGAGATTC	
45	PdSCPL41-ADF	GGAGGCCAGTGAATTCATGGATTTGGTACCTAAGGTG	
46	PdSCPL41-ADR	TCATCTGCAGCTCGAGTCTCACAGAAGGCAGGGATTC	
47	PdWND1B-ADF	GGAGGCCAGTGAATTCATGCCTGAGGATATGATGAATC	
48	PdWND1B-ADR	TCATCTGCAGCTCGAGTACCGATAAGTGGCATAATGG	
49	MAP2698-ADF	GGAGGCCAGTGAATTCATGGACCCTGCGAGTATACTTG	
50	MAP2698-ADR	TCATCTGCAGCTCGAGACTTCGTCTTTCTTCCTGTTTC	
51	KOR1-ADF	GGAGGCCAGTGAATTCATGAGCATGTATGGTAGAGATC	
52	KOR1-ADR	TCATCTGCAGCTCGAGTGGTCTCCAAGGTGCTGGAGGTG	
53	UGPase-ADF	GGAGGCCAGTGAATTCATGGCTACTGATACGGCCAAG	
54	UGPase -ADR	TCATCTGCAGCTCGAGGAGGTCCTCCGGGCCATTG	
55	CaM247-IF-ADF	GGAGGCCAGTGAATTCATGGCGGATCAATTGACGGATG	
56	CaM247-IF-ADR	TCATCTGCAGCTCGAGCTTAGCCATCATAACCTTGAC	
57	CaM1179IF-ADF	GGAGGCCAGTGAATTCATGGGCAAGGATCTGAGCGACG	
58	CaM1179IF-ADR	TCATCTGCAGCTCGAGCTTCGTAACCATTCTGGCAATAAAATC	
59	CaM-1249-ADF	GGAGGCCAGTGAATTCATGCCAACTATTTTGCTTAGG	
60	CaM-1249-ADR	TCATCTGCAGCTCGAGACCCCAAAGCACTAAGCCACCACCC	
61	CaM-351-ADF	GGAGGCCAGTGAATTCATGCAAACCGGGTATGTTGAGAG	
62	CaM-351-ADR	TCATCTGCAGCTCGAGAGAATCATCTAACTCTATGAGCTGTGCTC	
63	CaM-1281-ADF	GGAGGCCAGTGAATTCATGGCAACTGATAGAGTTTC	
64	CaM-1281-ADR	TCATCTGCAGCTCGAGGGCCATCATTTGATTAAACTCATGAAAATC	
65	CaM-1343-ADF	GGAGGCCAGTGAATTCATGGGGTTTAAATGCCTTTTC	
66	CaM-1343-ADR	TCATCTGCAGCTCGAGACCCCTAGTTCCTCTTAGAGTATC	
67	CaM-297-ADF	GGAGGCCAGTGAATTCATGCCAACTATTTTGCTTAGG	
68	CaM-297-ADR	TCATCTGCAGCTCGAGACCCAAAGCACTAAAGCCACCACCC	
69	CaM939-ADF	GGAGGCCAGTGAATTCATGGCATCGACGGTGATTGC	
70	CaM939-ADR	TCATCTGCAGCTCGAGGCTCGTATTGCCTAATATATATTTC	
71	CaM319-ADF	GGAGGCCAGTGAATTCATGGAAACTGAAAGAGCTTC	
72	CaM319-ADR	TCATCTGCAGCTCGAGAGCCATCATTTGATTAAACTC	
73	CaM437-ADF	GGAGGCCAGTGAATTCATGAAATTCATCAAAAAACTC	
74	CaM437-ADR	TCATCTGCAGCTCGAGTCTCCGCAGCTCCATCATAC	
75	CaM1229-ADF	GGAGGCCAGTGAATTCATGCACATCATTCATAATTTA	
76	CaM1229-ADR	TCATCTGCAGCTCGAGACAAAAACTGTTCTCCATGAAC	
77	CaM3149-ADF	GGAGGCCAGTGAATTCATGAAAGATTCACTTGGAAC	
78	CaM3149-ADR	TCATCTGCAGCTCGAGAGTTCTTGTGCTCACAGGAATATC	
79	CaM403-ADF	GGAGGCCAGTGAATTCATGGTGGATGTGTTTACAGAG	
80	CaM403-ADR	TCATCTGCAGCTCGAGAATTGCCAGCATCATTCTC	
81	CaM473-ADF	GGAGGCCAGTGAATTCATGGCAGATGCACTGGCTGG	
82	CaM473-ADR	TCATCTGCAGCTCGAGGAAAGCCATCGCCATCATCC	
83	CaM2391-ADF	GGAGGCCAGTGAATTCATGGATCCGGCAGAGCTACG	
84	CaM2391-ADR	TCATCTGCAGCTCGAGACTTGAACCTAGGGCAGCAAATC	
85	CaM326-ADF	GGAGGCCAGTGAATTCATGACGGAGCAGCTAACAGAG	
86	CaM326-ADR	TCATCTGCAGCTCGAGCTTGGCCAGCATCATCCTCAC	
87	CaM528-ADF	GGAGGCCAGTGAATTCATGGTAGATGTGTTGACGGAAG	
88	CaM528-ADR	TCATCTGCAGCTCGAGAGCAGCCAGCATTATTCTCAC	
89	CaM410-ADF	GGAGGCCAGTGAATTCATGTCTGAGCAGCTAACGGAG	
90	CaM410-ADR	TCATCTGCAGCTCGAGCTTGGCCAACATCATCCTCAC	
91	CaM215-ADF	GGAGGCCAGTGAATTCATGGCCGATCAGCTGACCG	
92	CaM215-ADR	TCATCTGCAGCTCGAGTAACACATTACAGCATTTTTTC	
93	CaM957-ADF	GGAGGCCAGTGAATTCATGAGCAACAAGCAACCAGC	
94	CaM957-ADR	TCATCTGCAGCTCGAGAACCCAAGAATTATCAAAAG	
95	CaM526-ADF	GGAGGCCAGTGAATTCATGACAGAGGCAGCCCTACATG	
96	CaM526-ADR	TCATCTGCAGCTCGAGAGTGATGCCTAAGAACTCC	
97	CaM014-ADF	GGAGGCCAGTGAATTCATGAAATCACACTCGGTTTC	
98	CaM014-ADR	TCATCTGCAGCTCGAGGCGCATCATGACAGCAAACTC	
99	CaM659-ADF	GGAGGCCAGTGAATTCATGATGATGATCATTACCATC	
100	CaM659-ADR	TCATCTGCAGCTCGAGGAAGACAGAAACAAGCTTGC	
101	CaM2599-ADF	GGAGGCCAGTGAATTCATGAAGTCACACTCAGTTTC	
102	CaM2599-ADR	TCATCTGCAGCTCGAGGCGCATCATGACAGAAAACTC	
103	CaM482-ADF	GGAGGCCAGTGAATTCATGGCAACCCCGAACGCCAAAAC	
104	CaM482-ADR	TCATCTGCAGCTCGAGGGAACCCATATTAGCGGCCATC	
105	CaM267-ADF	GGAGGCCAGTGAATTCATGGCGGATCAATTGACTGATG	
106	CaM267-ADR	TCATCTGCAGCTCGAGCTTAGCCATCATAACCTTGAC	
108	IQdom-BDF	CATGGAGGCCGAATTCGAATATATAGCTGCTGTCAGG	
109	IQdom-BDR	GCAGGTCGACGGATCCCCTCACGGCGCCCTTAAGACGG	
110	C014-pEF	CACCATGAAATCACACTCGGTTTCATC	pENTR gateway cloning
111	C014-pER	GCGCATCATGACAGCAAACTC	
112	C014-pER w stop	CTAGCGCATCATGACAGCAAACTC	
113	UBCc-F	CTGAAGAAGGAGATGACAGCAC	qPCR primers
114	UBCc-R	GCATCCCTTCAACACAGTTTCAC	
115	IQD10-RTF	TGGAGAGCCAACCCTACCCAATA	
116	IQD10-RTR	GCAGGTTTGGTTGGAAGTGTCGTT	
117	18S-F	AATTGTTGGTCTTCAACGAGGAA	
118	18S-R	AAAGGGCAGGGACGTAGTCAA	
119	PdWND1B-pX6p1F	GAAGGTCGTGGGATCCATGCCTGAGGATATGATGAATC	GST-fusion recombinant proteins
120	PdWND1B-pX6p1R	GTCGACCCGGGAATTCTACCGATAAGTGGCATAATGG	
121	PdHB3-6p1F	GAAGGTCGTGGGATCCATGGCGCTTTCTATGCACAG	
122	PdHB3-6p1R	GTCGACCCGGGAATTCCACAAAGGACCAGTTAATGAAC	
123	ProMYB002-F	ACCTCTCTCATTTTCCCCTGC	Promoter amplicons for EMSA assay
124	ProMYB002-R	TCCCTGTCACTAGAAAGGTGATCT	
125	ProHB3-F	GCCTGCCTCTCATTTATTCTCTAC	
126	ProHB3-R	CACCTAAAGAAAGAACTAAAACTTG	
127	ProIQD1-F	AAGTTAAAACGGTTGAAGATGG	
128	ProIQD1-R	GGTTCAAAGAGAGAAGCAACAC	
129	ProCaM014-F	CCGGAAATGCCGAGAAGGACTCG	
130	ProCaM014-R	AAAGGAAATGAGGGTGTGGTCATGGC	
131	ProAct-F	ACCTACTTCGTTTGGTCATTGTTA	
132	ProAct-R	CAAATACAACATACTAGTTCCTCCAC	


### Plant Growth and Sampling

Transgenic and empty vector transformed control plants were acclimated from the tissue culture to Ray Leach tubes containing equal parts Fafard 52:perlite:vermiculite. After 2 months, the plants were moved to bigger pots (6 l) and propagated in a greenhouse maintained at 25°C with 16 h day length under drip irrigation and fertilized weekly with 200 ppm N. At the time of harvest (7-month old plants), plant height was measured from shoot tip to stem base, and diameter was measured at ∼5 cm from the soil line. In our preliminary study, the basal, lignified 10 cm stem portion was harvested, debarked, air-dried, and used for carbohydrate composition, cellulose, lignin, S:G ratio, and sugar analyses. Initial studies were performed on 18 transgenic lines (plus 9 control lines) and additional studies were performed on three to four selected lines. Plants for additional studies were generated from fresh internodal stem cuttings. The tissues collected were young leaves (leaf plastochron index, LPI-0 and 1), mature leaves (LPI-6) and stems (internode portion between LPI 6 and 8), which were frozen in liquid nitrogen and stored at -80°C until used.

For tension stress response study, *Populus deltoides* plants were grown erect as control or with bending stress to generate tension wood on the outer bent side and opposite wood on the inner stem side as follows. The six plants per treatment were grown from stem cuttings rooted and grown in the greenhouse for ∼40 days prior to start of the experiment. Plants were tied in a bent position to induce tension wood formation and the control plants were left straight but were also tied to stakes to simulate the same thigmotactic set-up as the plants under tension, for a 2-week period of time. Xylem tissue was collected from freshly harvested samples, flash-frozen and processed as previously described (Kalluri et al., 2009).

### RNA Extraction and Gene Expression Studies

RNA from the ground and frozen stem samples was extracted using a Plant RNA extraction kit (Sigma, St. Louis, MO, United States), with modifications. Briefly, 100 mg of frozen sample was extracted with a 850 μl CTAB buffer maintained at 65°C followed by chloroform:isoamylalcohol (24:1 v/v). After passing the supernatant through a filtration column, the eluent was diluted with 500 μl of 95% EtOH and passed through a binding column. Further steps, including on-column DNAse digestion, were followed as per the manufacturer protocol. cDNA was synthesized from 1.5 μg of RNA using oligo dT primers and RevertAid Reverse Transcriptase (Thermofisher). Quantitative reverse transcriptase PCR (qRT-PCR) was performed in a 384 well plate using cDNA (3 ng), gene specific primers (250 nM, list provided in Table 2) and iTaq Universal SYBR Green Supermix (1X, Bio Rad). Gene expression was calculated by a ΔC*_T_* or ΔΔC*_T_* method using the expression of housekeeping genes 18S ribosomal RNA and Ubiquitin-conjugating enzyme E2 for template normalization (Gene accession numbers and primer sequence information can be found in Payyavula et al. (2014).

### Cellulose and Lignin Properties

The cellulose content in an air-dried stem sample was estimated using the anthrone method (Updegraff, 1969) as well as wet chemistry-based HPLC analysis. For the semi-quantitative anthrone assay, stem sample (25 mg) was first digested with 500 μl of acetic-nitric acid reagent (100 ml of 80% acetic acid mixed with 10 ml of nitric acid) at 98°C for 30 min. After cooling, the sample was centrifuged. The supernatant was discarded and the sample was washed with water. After a brief centrifugation, the water was removed and the pellet was digested with 67% (v/v) sulfuric acid for 1 h at room temperature. An aliquot of the mix was diluted (1:10) with water. In a PCR tube, 10 μl of diluted reaction mix, 40 μl of water and 100 μl of freshly prepared anthrone reagent (0.5 mg anthrone ml^-1^ of cold concentrated sulfuric acid) were added and heated for 10 min at 96°C. The samples were cooled and the absorbance (A_630_) was measured. The cellulose content was then estimated based on the absorbance of glucose standards. Holocellulose and α-cellulose samples were prepared and employed in gel permeation chromatography (GPC) and for a ^13^C-CPMAS NMR analysis of cellulose using established protocols.

For quantitative wet chemistry assay, roughly 25 mg of air-dried stem sample was weighed in a 2 ml tube and twice extracted at 85°C to a total of 2 ml of ethanol (80%). To eliminate pigments that interfere with sugar analysis, the supernatant was collected in a new 2 ml tube and re-extracted with 50 mg of activated charcoal (Sigma). A 1-ml aliquot of the pigment-free extract was incubated overnight in a heating block maintained at 50°C. The resulting pellet was dissolved in 120 μl of water and a 10 μl aliquot was used in sucrose and glucose estimation using kits (Sigma). Starch from the pellet was digested by 1 U of α-amylase (from *Aspergillus oryzae*, Sigma) and amyloglucosidase (from *Aspergillus niger*, Sigma). After starch removal, the pellet was dried overnight at 95°C and used to estimate structural sugars. Roughly 5 mg of sample was weighed in a 2-ml tube and digested with 50 μl of 75% v/v H_2_SO_4_ for 60 min. The reaction was diluted by adding 1.4 ml water. The tubes were sealed using lid-locks and autoclaved for 60 min in a liquid cycle. After cooling, the sample was neutralized with CaCO_3_ and sugar composition was estimated with high performance liquid chromatography (HPLC, LaChrom Elite^®^ system, Hitachi High Technologies America, Inc.), as described previously.

Lignin content and the syringyl-to-guaiacyl ratio (S/G ratio) were determined based on pyrolysis molecular beam mass spectrometry (MBMS) of dried and ground stem biomass samples as described previously (Mielenz et al., 2009; Kalluri et al., 2016).

### *In silico* Identification of the PdIQD10 Interacting Partners

Putative interacting partners of PdIQD10 (Table 3) were identified using various *in-silico* databases like String (using POPTR_0001s38470 as query)^1^, ATTED-II^2^ and phytozome co-expression database^3^. For the *Arabidopsis* genes identified using ATTED-II, the closest homolog of *Populus* was chosen as a putative interacting partner. The genes *GAUT12.1* (Biswal et al., 2015; Potri.001G416800.1), *IRX10* (Porth et al., 2018; Potri.001G068100.1), *PdSCPL14* and *PdSCPL41* (Zhu et al., 2018; Potri.004G037800.1; Potri.011G046600.1) were named according to the respective *Populus* literature. The genes *DICE1* (Le et al., 2018; Potri.006G269800.1), *DUF1218* (Wilson-Sánchez et al., 2017; Potri.004G235000.1), *CHUP1* (Oikawa et al., 2008; Potri.001G279000.1), *RIC4* (Gu and Nielsen, 2013; Potri.002G233400.1); *UC2* (Xu et al., 2017; Potri.002G101300.1); *Lac17* (Voxeur et al., 2017; Potri.001G401300.1), *NEK5* and *NEK6* (Vigneault et al., 2007; Potri.016G051900.1; Potri.006G056300.1) were named according to the Arabidopsis convention reflecting their closest homologs. The names of three Microtubule Associated Proteins - MAP599, MAP1733 and MAP2698 were based on (Quentin et al., 2016) with the suffix numbers corresponding to the last four digits of respective gene IDs.

**Table 3:** List of putative interacting partners tested.

Sl. No	Potri ID	Gene names	Putative function (At orthologs)	Prediction database
1	Potri.T059900.1	MAP599	Microtubule-associated protein	ATTED co-expression
2	Potri.014G108300.1	DICE1	DEFECT IN CELL ELONGATION1	
3	Potri.010G141600.1	Chi1416	Chitinase-like protein 2	
4	Potri.004G235000.1	DUF1218	Transmembrane protein with unknown function	
5	Potri.001G279000.1	CHUP1	Chloroplast unusual positioning1	
6	Potri.002G233400.1	RIC4	Cdc42- and Rac-interactive binding (CRIB) motif-containing protein	
7	Potri.001G231600.1	HYP2316	Similar to hypothetical protein with unknown function	
8	Potri.002G101300.1	UC2	Plastocyanin-like domain-containing protein	
9	Potri.001G401300.1	Lac17	Laccase-like	
10	Potri.006G181900.1	PtiCesA7-A	Similar to cellulose synthase	Phytozome co-expression
11	Potri.003G173300.1	MAP1733	Microtubule-associated protein	
12	Potri.001G416800.1	PdGAUT12.1	Galacturonosyltransferase 12-related	
13	Potri.001G068100.1	IRX10	beta-1,4-xylosyltransferease	
14	Potri.016G051900.1	NEK5	Serine/Threonine-protein kinase	STRING
15	Potri.006G056300.1	NEK6	Serine/Threonine-protein kinase	
16	Potri.004G037800.1	PdSCPL14	Serine carboxypeptidase	
17	Potri.011G046600.1	PdSCPL41	Serine carboxypeptidase	
18	Potri.001G448400.1	PdWND1B	NAC-domain transcription factor	Genes with cell-wall related functions
19	Potri.006G269800.1	MAP2698	Microtubule-associated protein	
20	Potri.001G078900.1	KOR1	Korrigan	
21	Potri.017G144700.1	UGPase	UDP-glucose pyrophosphorylase	


Calmodulin (CaM) and calmodulin-like (*CML*) genes encoded by the *Populus* genome were identified by repeated BLAST searches in the Phytozome database using *Arabidopsis* CaM/CML protein sequences as query. The identified proteins were named as they appear in Table 1 in which the numbers following the CaM symbol are derived from their respective Potri IDs. The amino acid sequences of *Arabidopsis* and *Populus* CaM/CML proteins were used to build a phylogenetic tree using Maximum Likelihood method of the MEGA 7.0.25 software.

### Yeast Two-Hybrid Assay

The coding sequences of the 21 putative interactors and 26 calmodulin/calmodulin-like CaM genes were cloned into pGADT7 (Clontech) vector and *PdIQD10* into pGBKT7 (Clontech) vector using In-Fusion^®^ Advantage PCR Cloning Kit (Clontech). The primers used for cloning are listed in Table 2. The generated constructs were transformed into yeast Y2H Gold (Clontech) competent cells using Fast^TM^ Yeast Transformation (G-Biosciences Cat. #GZ-1) kit. Transformed yeast cells were plated on Yeast Synthetic Drop-out (SD) media lacking Leucine and Tryptophan amino acids (Sigma–Aldrich #Y0750) to select the positive transformants for both plasmids. At least two clones were individually tested either on SD media lacking histidine, leucine, and tryptophan (Sigma–Aldrich #Y2146) or on SD media lacking histidine, leucine, tryptophan and adenine (Sigma–Aldrich #Y2021) in three different transformation experiments to determine the interaction result.

### β-galactosidase Assay

The strength of the observed interactions was assessed by quantifying the activity of β-galactosidase using ONPG substrate as described in the Yeast Protocols Handbook (Clontech). Briefly, overnight yeast cultures in SD selection medium are inoculated in YPD medium to grow until mid-log phase (OD600 of 1 ml = 0.5–0.8). Yeast cells were harvested, washed once in Z-buffer (40 mM Na_2_HPO_4_.7H_2_O, 60 mM NaH_2_PO_4_.7H_2_O, 10 mM KCl, 1 mM MgSO_4_.7H_2_O, pH 7.0) and subjected to repeated freeze-thaw cycles to break open yeast cells. The lysate is then supplemented with Z-buffer, β-mercaptoethanol and ONPG (final ∼100 μg) and incubated at 30°C for 24 h. The reactions were stopped by adding Na_2_CO_3_ and the absorbance was measured at OD_420_. β-galactosidase activity was calculated by using the equation: β-galactosidase units = 1,000 × OD_420_/(t × V × OD_600_). The assay was performed three times using yeast transformants from three independent transformation experiments.

### Structural Modeling/Molecular Dynamics Simulation (MD)

Structural models of PdIQD10 (Potri.001G375700.1), six CaMs (Potri.016G024700.2, Potri.002G001400.1, Potri.001G222200.1, Potri.006G026700.1, Potri.009G021500.1, Potri.012G041000.1) and an PdIQD10-domain (Potri.001G375700.1) models were built using the iterative threading assembly refinement (I-TASSER, version 5.0) (Roy et al., 2010) protein structure modeling toolkit. A 200-ns molecular dynamics (MD) simulation was performed on the complex formed by the PdIQD10-domain and calmodulin. For the MD simulation a water box with at least 15 Å to the edge of the protein was used, and sodium/chloride ions were added to balance the net charge of the whole system. The software NAMD (Phillips et al., 2005) was used for the MD simulation. The CHARMM protein force field (Best et al., 2012) and TIP3P water model (Jorgensen et al., 1983) were adopted in all MD simulations. A time step of 2-fs was applied with the SHAKE algorithm to fix the bonds involving hydrogen atoms. In the MD simulation, after a 50,000 steps energy minimization, the temperature of the system was gradually heated to 300 K with a rate of 0.001 K per time step. The MD simulations were performed under an NPT ensemble with the system pressure of 1 atm and temperature of 300 K maintained by the Langevin piston controls. Cutoff of switching between 9 and 11 Å was applied for the non-bonded interactions, and particle mesh Ewald summation with a grid spacing of 1.35 Å were applied for long range electrostatic interactions, respectively.

### *In vitro* Promoter Binding Experiments

Full-length *PdHB3* (Potri.011G098300) and *PdWND1B* (Potri.001G448400) were cloned in pGEX-6P-1vector and GST-fused recombinant proteins were isolated and used for the assays. Promoter regions representing 250 bp upstream of transcriptional start sites in genomic sequences of *PdHB3* (Potri.011G098300), *PdIQD10* (Potri.001G375700) and *PdCaM014* (Potri.002G001400) and *PdMYB002* (Potri.001G258700) genes were used for the assays. Electrophoretic Mobility Shift assays (EMSA) were performed using Thermo Scientific LightShift^TM^ Chemiluminescent EMSA Kit according to manufacturer’s instructions. Briefly, ∼2 picomoles of the amplified promoter regions were biotin labeled using Pierce^TM^ Biotin 3′ End DNA Labeling Kit. Biotin labeled fragments (∼100 femtomoles) were incubated with 200–300 nanograms of the GST-fusion recombinant proteins in the reaction buffer (1X Binding buffer (20148A), 2.5% glycerol (20148F), 5 mM MgCl_2_ (20148I), 50 ng/μl poly dI:dC (20148E), 0.05% NP-40 (20148G)) for 20 min at room temperature. For competition assays, ∼200 fold-excess of the respective unlabeled DNA fragments were used. The reactions were separated on 6% DNA Retardation Gels (EC6365BOX), transferred on to nylon membrane and crosslinked at 120 mJ/cm2 using ULTRA-LUM UVC 515 Ultraviolet Multilinker for 60 s. The membrane was then followed with the detection procedure of the kit and the chemiluminescence was detected using BioRad ChemiDoc^TM^ XRS+ System.

### Transcriptional Activator Assay

The coding sequences (CDS) of *PdIQD10*, and four *KLC* homologs; *KLC400*, *KLC3200*, *KLC4700*, and *KLC7800* were in-frame cloned in Gal4 binding domain (GD) effector vector (Wang et al., 2007). For the trans activator assays, the GD-fusion constructs were co-transfected with *Gal4*:*GUS* reporter construct into *Populus* 717 protoplasts (Guo et al., 2012). Empty GD effector vector was co-transfected with reporter vectors for the control experiments. The transfected protoplasts were incubated in dark for 16–20 h and GUS activity was quantitatively measured. All the protoplast transfections were included with equal amounts of 35S:Luciferase reporter construct and Luciferase activity was used for normalization of GUS activity. The quantification of GUS and Luciferase were performed as below.

For, quantitative measurements of β-glucuronidase and Luciferase, transfected protoplasts were lysed using 1X Cell Culture Lysis Reagent (Promega Cat. # E1531) followed by incubation on ice for 5 min. Cell-debris was separated by centrifugation at 2000 rpm for 3 min and the supernatant was used for the assays. For GUS activities, equal amounts of cell lysate was incubated with 1X solution of 4-methylumbelliferyl β-D-glucuronide (MUG) in the reaction buffer [10 mM Tris (pH 8.0), and 2 mM MgCl_2_] at 37°C for 1 h. The reactions were stopped by adding 0.2 M Na_2_CO_3_ and fluorescence was measured at 460 nm when excited at 355 nm. For luciferase activities, cell lysate was mixed with Luciferase Assay Reagent (Promega Cat. # E1500) and the luminescence was measured. Both fluorescence and luminescence were measured using BioTek^TM^ Synergy^TM^ 2 Multi-Mode Microplate Reader.

## Results

### *PdIQD10* Gene Belongs to the IQ 67-Domain Containing IQD Family and Shows Enhanced Expression in the Context of Secondary Cell Wall Biosynthesis

**FIGURE 1:**
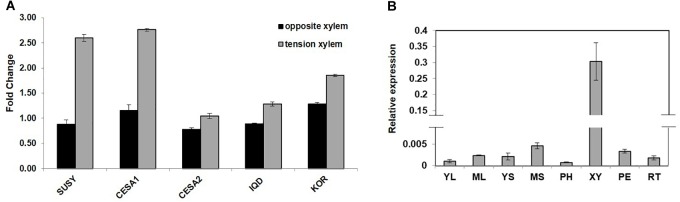
**(A)** Expression profile of cell wall related genes in tension stressed and opposite xylem tissue. qRT-PCR data showing the relative transcript levels of the chosen genes involved in cell-wall formation. Known cellulose pathway marker genes such as sucrose synthase (*SUSY; Potri.006G136700*) cellulose synthases (*CESA1; Potri.004G059600, PdCESA8-B; CESA2; Potri.018G103900, PdCesA7-B)*, and *KOR* (Potri.001G078900) display higher expression in the tension stressed xylem tissue along with *IQD* (*PdIQD10*; Potri.001G375700) gene as compared to the opposite xylem. **(B)** Transcript profiling of *PdIQD10* in various tissues of *Populus*. The expression levels of *PdIQD10* was determined by qRT-PCR method using *Populus cDNA* libraries of Young Leaf (YL), Mature Leaf (ML), Young Stem (YS), Mature Stem (MS), Phloem (PH), Xylem (XY), Petiole (PE) and Root (RT). As shown in the figure, the expression of *PdIQD10* transcript is highest in xylem tissue followed by mature stem. Standard deviation was calculated across biological replicate libraries (*n* = 3). A break in *Y*-axis denotes discontinuity in scale. The corresponding figure with continuous Y-axis scale is provided as Supplementary Figure S9. Relative expression was based on changes in critical threshold (R_C_RT) values relative to housekeeping genes.

**FIGURE 2:**
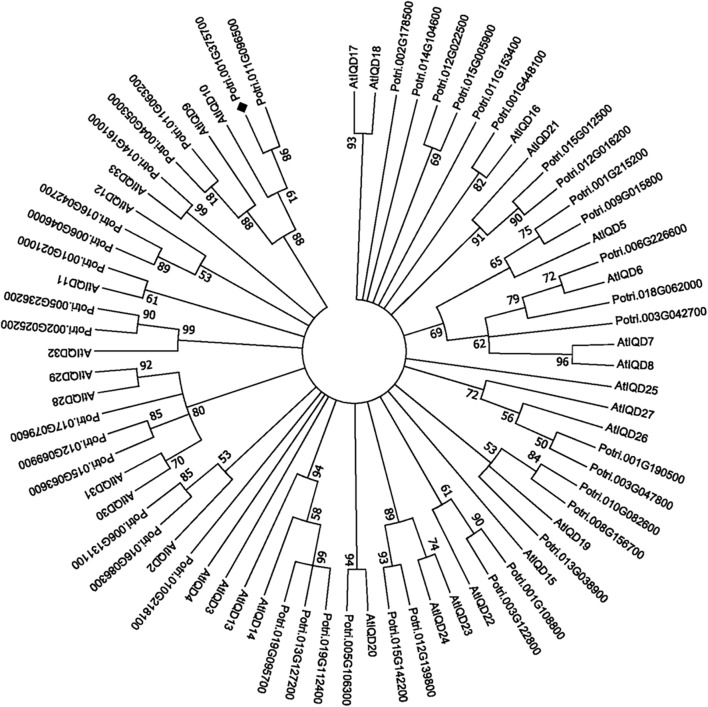
Phylogenetic analysis of *IQD* gene family members from *Populus* and *Arabidopsis*. The predicted IQD protein sequences from *Populus* and *Arabidopsis* were used to generate the phylogenetic distance tree. The tree was generated using Maximum Likelihood algorithm in MEGA7.0.25 with 1000 bootstrap replicates and represents 42 *Populus* and 33 *Arabidopsis* IQD members. The target of this study, PdIQD10 (Potri.001G375700), is marked “
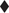
”.

*PdIQD10* (Potri.001G375700), was identified from our previously undertaken studies including; tension stress response characterization, proteomics of developing xylem, and co-expression network analysis of *Populus* and *Arabidopsis* stem tissues (Kalluri et al., 2009; Yang et al., 2011; Abraham et al., 2012). Gene-specific qRT-PCR assays undertaken to support the findings of these three studies confirmed the enhanced expression of *PdIQD10* gene in tension-stressed xylem and secondary walled cells (Figure 1A). Among the native tissues profiled including leaf, stem and root tissues, *PdIQD10* showed a significantly higher expression in the xylem tissue (Figure 1B). *PdIQD10* expression data from the recent transcriptome study by Shi et al. (2017) is in agreement with our findings showing higher *PdIQD10* expression in xylem and fiber libraries (Supplementary Figure S2). Sequence analysis suggests that *PdIQD10*, codes for a predicted calmodulin-binding protein belonging to the family IQ67-domain containing proteins IQD (referenced after the isoleucine and glutamine (IQ)- amino acid rich region and the central domain of 67 conserved amino acid residues). A BLASTP search of the Phytozome database (*Populus* genome v. 3.0) using 33 *Arabidopsis* IQD protein sequences as query identified a total of 42 PtIQDs. The phylogenetic tree was constructed with predicted PtIQD and AtIQD protein sequences using a Maximum Likelihood algorithm in MEGA7.0.25 with 1000 bootstrap replicates (Figure 2). The present manuscript describes the functional characterization of a corresponding *P. deltoides* ortholog, *PdIQD10*, of *PtIQD10* gene (Potri.001G375700 in the reference *P. trichocarpa* genome).

### PdIQD10 Is a Calmodulin-Binding Protein

**FIGURE 3:**
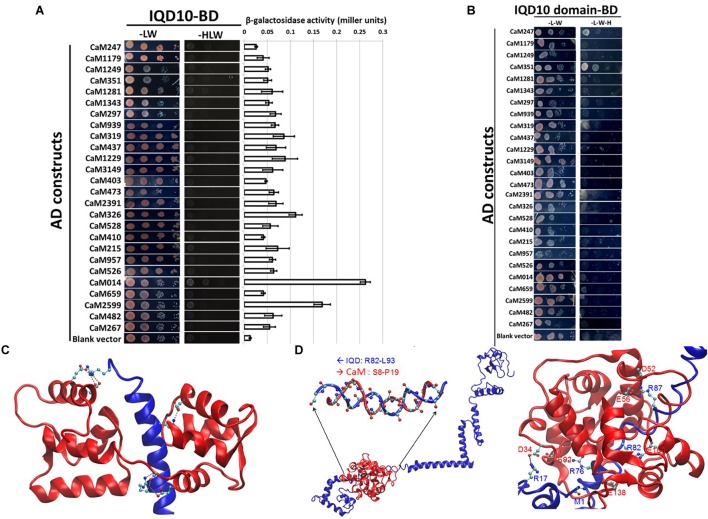
Yeast two-hybrid interaction analysis of PdIQD10 with CaMs. **(A)** Yeast two-hybrid interaction analysis of full-length PdIQD10 with 26 calmodulins encoded by *Populus* genome. This figure shows the positive interaction of PdIQD10 with CaM014 and CaM2599. Bar graph on the right represents the average β-galactosidase activities assayed from three independent yeast colonies to determine the strength of the interactions. **(B)** Yeast two-hybrid interaction analysis of IQ67-domain of PdIQD10 with different calmodulins. The IQ67-domain only version of PdIQD10 interacts with CaM247 and CaM351 but not with CaM014 and CaM2599 indicating the difference in functionalities and specificities between full-length PdIQD10 and IQ-67 domain only version. **(C)** A cartoon representation of MD simulation showing IQD-domain only and CaM247 protein interactions. The flip-and-flop of Calmodulin (CaM, in Red) around the IQD domain (in Blue) makes many of the interactions between the two molecules transient. Strong electrostatic interactions (i.e., salt bridges) persist. **(D)** A cartoon representation of the complex of full-length IQD (blue) and CaM014 (red); (left) the inset shows a hybrid duplex between R82 to L93 of IQD and S8 to P19 of CaM mainly formed by dipole-dipole interactions between the backbone of the regions of both proteins; (right) Salt-bridge interactions between the basic residues (Arg and the backbone of N-terminus) of IQD and acidic residues (Asg and Glu) of CaM with basic residues.

The calmodulin binding ability of targeted *Arabidopsis* IQ67-domain-containing proteins has been previously demonstrated (Levy et al., 2005; Abel et al., 2013; Bürstenbinder et al., 2013). To test the predicted ability of PdIQD10 to bind a calmodulin protein, calmodulin/calmodulin-like genes encoded by *Populus* genome were identified using Phytozome^4^ database. Table 1 lists 36 calmodulin and calmodulin-like *CaM* genes their inclusion in the yeast two-hybrid assay and their corresponding *Arabidopsis* homologs. *PdIQD10* was cloned into pGBKT7 vector and was used as a bait to identify interactions with the full-length *CaM* cDNAs cloned in pGADT7 vector in targeted one-to-one yeast two-hybrid assays. Among a total of 26 interactions screened, CaM014 and CaM2599, induced the growth of yeast auxotroph colonies on minimal media lacking histidine, leucine and tryptophan indicating weak positive interactions (Figure 3A). The quantification of interaction strengths by β-galactosidase activity add support to the weak interaction inferred from slow colony formation. Furthermore, although CaM014 and CaM2599 share 79.2% identity in their protein sequences (Supplementary Figure S3), interaction with CaM014 was found to be stronger relative to that with CaM2599. *In vitro* studies undertaken by Bürstenbinder et al. (2013) suggest that IQ67-domain is the calmodulin binding region of IQD proteins. To investigate this prediction and the importance of the region flanking IQ67 domain for interaction with CaMs, IQ67-domain of PdIQD10 (PdIQD10-domain) was cloned into pGBKT7 vector and used as a bait with CaMs. Contrary to theoretical expectation that IQ67-domain-version can interact with most CaMs, specificity in interaction was observed. CaM247 and CaM351 were found to interact with IQ67-domain of PdIQD10 whereas CaM014 and CaM2599 were found not to interact (Figure 3B). The observed specificity of IQ67 domain-only version is in contrast with that observed for the full-length version of the IQD, may suggest that IQ67-domain functions differently than the PdIQD10 full length protein. It is interesting to learn that the full-length PdIQD10 and PdIQD10-domain have non-overlapping calmodulin interacting partners. One reason for the discrepancy may be the differences involved in physiologies of yeast and that of the plant. Alternatively, the longer linker may translate into a 3-D structural conformational change that determines specificity. The sequence alignments of CaM247, CaM014 and CaM2599 protein sequences showed higher similarity between CaM014 and CaM2599, than with CaM247, which is shorter at its N-terminal (Supplementary Figure S4). This observation is also consistent in phylogenetic tree of CaM/CaM-like proteins where CaM014 and CaM2599 are in a single clade (Supplementary Figure S5).

A complementary computational approach based on molecular dynamics (MD) simulations was undertaken to probe the potential interaction sites between IQD and CaM proteins. MD simulations showed that the predicted transient interaction of IQD-domain only with CaM247 is stabilized in the presence of salt (Figure 3C). Specifically, the flip-and-flop of CaM around the IQD domain makes many of the interactions between the two molecules transient (Figure 3C). However, there are several strong electrostatic interactions (i.e., salt bridges) that persist throughout the MD simulations, including specific amino acid positions within CaM247 and IQD-domain only; E15-R29, E12-R29, E85-R7, D81-R7, and E121-K16. In addition to salt-bridge interactions, MD simulations showed the potential for a hybrid duplex structure interaction between full-length PdIQD10 (R82 to L93) and CaM014 (S8 to P19) (Figure 3D). Salt-bridge interactions between the basic residues (Arg and the backbone of N-terminus) of PdIQD10 and acidic residues (Asg and Glu) of CaM014, are noted as; M1 -E138, R17-D34, R87-E56, R87-D52, R82-E160 and R76-E92.

### PdIQD10, CaM014 and CaM247 Co-localize in Similar Subcellular Compartments

**FIGURE 4:**
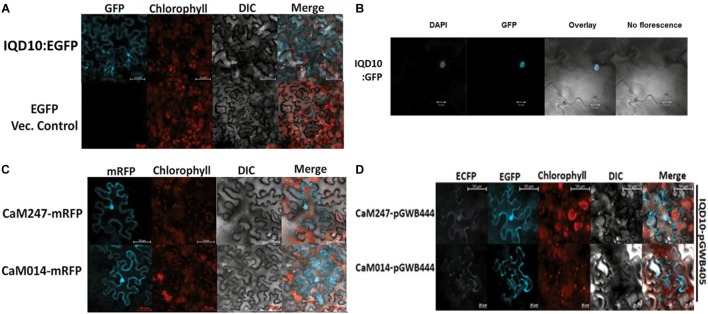
Subcellular localization of PdIQD10, CaM247 and CaM014. **(A)** Subcellular localization of PdIQD10-GFP was detected in the cytoplasm and plasma membrane. The fluorescence was detected uniformly throughout the cell post 72 h of agroinfiltration of tobacco leaves with the *Agrobacterium* clone containing PdIQD10 in pGWB405 vector. **(B)** Repeated assays detected PdIQD10-GFP localization in the nucleus of tobacco leaves agroinfiltrated with the clone harboring the same construct PdIQD10 in pGWB405. **(C)** CaM247-mRFP and CaM014-mRFP were found to be localized throughout the tobacco cells and in nucleus upon agroinfiltration of clones harboring CaMs in pGWB454 vector. **(D)** Co-localization of PdIQD1-EGFP with CaM247-CFP or CaM014-CFP was performed by mixing the *Agrobacterium* cultures in equal (1:1) ratio before agroinfiltration into tobacco leaves. To increase accessibility of these subcellular localization images, the yellow color channel was converted to magenta uniformly across all images in the CMYK color spectrum. The original RGB color scheme images are provided in Supplemental Figure 10. The color scheme is as follows: GFP/RFP: blue/cyan; chlorophyll, FM64 and mCherrry: red/orange; DAPI/CFP: gray/white.

Subcellular protein localization experiments were undertaken to understand the possible proximity and biological relevance of the observed interactions and its impacts on biomass properties of *Populus*. Fluorescent signal was observed under a confocal microscope, 72 h after an *Agrobacterium* clone carrying full length *PdIQD10* in pGWB405 vector was infiltrated in 4–6-week-old leaves of *Nicotiana benthamiana* (*N. benthamiana*). The signal from GFP tagged-PdIQD10 was detected in the cytoplasm and plasma membrane (Figure 4A). However, repeated localization experiments revealed that PdIQD10 also localizes to the nucleus (Figure 4B). CaM247 and CaM014 were cloned in-frame in pGWB454 vector expressing mRFP fusion tag. The fluorescence from both CaMs was also detected from nucleus and the plasma membrane further supporting their associated functions with PdIQD10 (Figure 4C and Supplementary Figure S6). PdIQD10-GFP co-localizes with CaM247-mRFP and CaM014-mRFP in the nucleus and in the plasma membrane supporting spatio-temporal co-localization patterns of the signaling molecules (Figure 4D). Similar protoplast assays under plasmolysis conditions will further confirm the localization in plasma membrane.

### PdIQD10 Is Potentially Regulated by Secondary Cell Wall Transcription Factors

**FIGURE 5:**
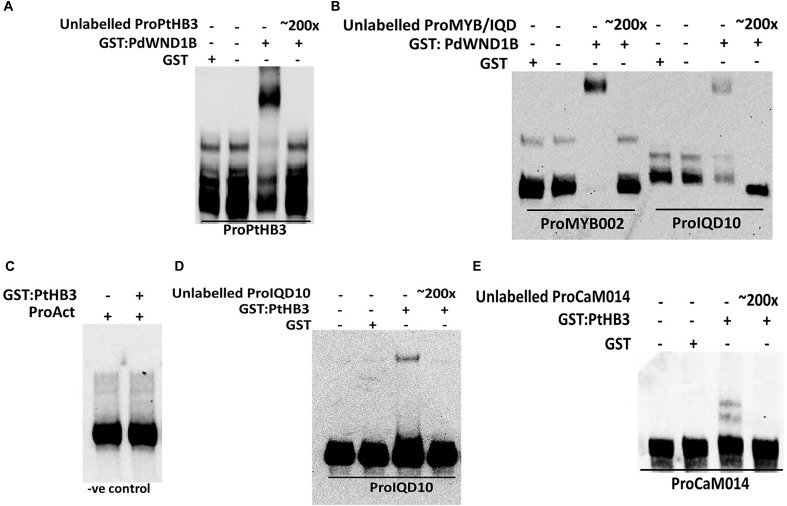
Electrophoretic Mobility Shift assays. GST fused protein of PdWND1B binds to the promoters of **(A)** PdHB3 and **(B)** Myb002 and IQD10. GST fused PdHB3 protein binds to the promoters of **(D)** PdIQD10 and **(E)** CaM014 but not to **(C)** Actin, suggesting a gene regulatory network downstream of PdWND1B. Promoter regions corresponding to the 250 bp upstream of the transcriptional start site of the respective genes were used for biotin labeling and shift experiments.

In order to understand the functional context of expression of *PdIQD10* gene in secondary wall forming cells, we undertook promoter binding assays using the secondary cell wall pathway transcription factors, PdWND1B, a *Populus* ortholog of the known master regulator of secondary cell wall biosynthesis in *Arabidopsis*, SND1 NAC domain transcription factor (Zhong et al., 2006; Zhao et al., 2014), and *PdHB3*, belonging to the HD-ZIP III family of transcription factors with known roles in stem development (Du et al., 2011; Robischon et al., 2011; Zhu et al., 2013). Electrophoretic Mobility Shift assays (EMSA) using GST-tagged fusion protein revealed that PdWND1B binds to the promoter of *PdHB3* (Figure 5A). Binding of PdWND1B to the *PdIQD10* promoter was observed to be weak compared to the binding to known positive control of *MYB002* promoter (Lin et al., 2013) (Figure 5B). PdHB3 was observed to have moderate binding affinity to the promoters of *PdIQD10* and its interacting partner *CaM014* and the binding is competed out upon inclusion of unlabeled promoters (Figures 5C–E). These experiments suggest that the *Populus* SND1 ortholog, *PdWND1B*, is regulating a transcriptional network that includes direct regulation of *PdHB3*, which in turn may regulate *PdIQD10* and *CaM014*.

### PdIQD10 Interacts With KLCR Proteins

**FIGURE 6:**
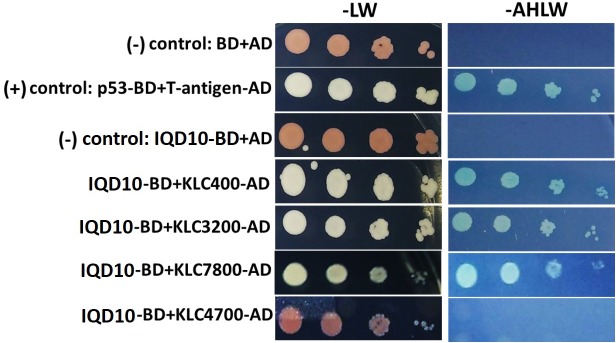
Yeast two-hybrid interaction analysis of PdIQD10 with Kinesin light chain related 1 (KLCR) **proteins.** Interaction analysis of PdIQD1 with four chosen KLC proteins using yeast two-hybrid approach. PdIQD1 interacts with KLC400, KLC3200 and KLC7800 but not with KLC4700. Positive control and the empty vector negative control are also shown.

Potential new pathway players and interactor proteins can be represented among tightly co-expressing gene sets. In order to identify new protein interacting partners for PdIQD10, we queried co-expression and protein interaction databases for identifying potential interactors in the context of stem development and cell wall biosynthesis. Selected top hits that were identified from *Populus* STRING^5^, *Arabidopsis* ATTED II^6^ and *Populus* Phytozome co-expression databases were employed in yeast two-hybrid assays (Table 3). The first set of proteins included sequence homologs of KLCR or KLC, a motor protein involved in unidirectional cargo transport along the microtubular network, which has previously been shown to interact with *Arabidopsis* IQD1 in a yeast two-hybrid cDNA library screen (Bürstenbinder et al., 2013). To test the functional conservation of PdIQD10 in *Populus*, we studied the interaction between PdIQD10 and four xylem-expressing KLCR-1 or KLC isoforms in *Populus*, *viz*., KLCR400 (Potri.014G100400), KLCR3200 (Potri.003G143200), KLCR7800 (Potri.001G087800) and KLCR4700 (Potri.008G094700) using yeast two-hybrid assays (Figure 6). These results suggest that three of the four KLCR proteins tested, KLCR400, KLCR3200 and KLCR7800, interact strongly with PdIQD10 (Figure 6). Domain structures of KLCR proteins shows a distinct primary protein structure for the fourth KLCR isoform, KLCR4700, which has an extended N-terminal region and a discontinuous tetratrico peptide repeat (TPRs) region at the N-terminal (Supplementary Figure S7). The C-terminal regions of these four KLCR proteins are similar in their domain architectures. It is plausible that interactions of PdIQD10 with specific KLCR proteins may be determined by variations in the N-terminal domain structure. Observed interaction of PdIQD10 with three out of four tested KLCRs indicates two things: first, PdIQD10 may interact with specific KLCRs and second, PdIQD10 might act as a cargo or a cargo associated (molecular scaffold) protein similar to the AtIQD1. Furthermore, the localization of PdIQD10 to the plasma membrane is in agreement with the understanding that cargos along the microtubules are generally transported by kinesins to the cell-periphery (Hirokawa et al., 2009; Verhey et al., 2011).

Additional potential interactor proteins were picked from publicly available co-expression databases. Selected proteins included in yeast two hybrid assays fell into the known or putative functional categories of cell wall formation (such as, cellulose synthase: PtiCesA7-A, Galacturonosyltransferase: GAUT12.1, glycosyltransferase family 47 member involved in xylan backbone synthesis; IRX10), cytoskeletal rearrangement (Microtubule associated proteins: MAP599, MAP1733, MAP2698), vascularization (NIMA related kinases: NEK5, NEK6) and/or transcriptional regulation of secondary cell wall formation (NAC domain transcription factor, PdWND1B), which are aligned with the functional context of PdIQD10 proposed here. Based on measured β-galactosidase activity, NEK6, SCPL14 and SCPL41 (Serine carboxypeptidase-like), appear to display weak interactions with PdIQD10 while assays with other proteins showed no interaction with PdIQD10 (Supplementary Figure S8).

### *PdIQD10* RNAi-Downregulated Transgenic Lines Show Altered Biomass Properties

**FIGURE 7:**
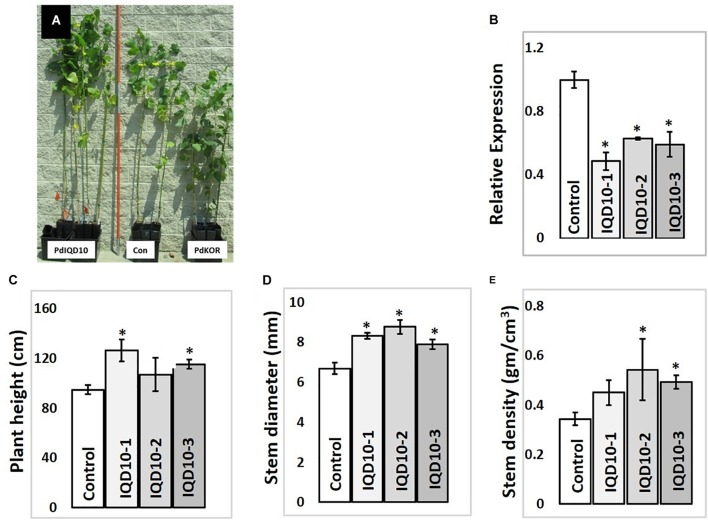
Growth characterization of *PdIQD10* RNAi lines **(A)** 6-month -old greenhouse grown transgenic plants representing *IQD* RNAi (*PdIQD10-1*), empty vector control and a comparator reduced biomass line *PdKOR* RNAi (Kalluri et al., 2016). **(B)** qRTPCR validation of RNAi-mediated downregulation of *PdIQD10* gene in three independent RNAi lines. **(C)** Measurements of average plant height, **(D)** stem diameter and **(E)** stem density of *PdIQD10* RNAi lines in comparison with that of vector control lines. Data represent means ± SE (*n* = 3–5). ^∗^Indicates statistically significant, *p* ≤ 0.05 based on Student’s *t*-tests.

In order to evaluate the functional role of *PdIQD10* in stem development and chemistry, transgenic *Populus deltoides* plants with stable, RNAi-mediated, downregulation of the *PdIQD10* gene were generated (Figure 7B). Preliminary growth assessment of 6-month-old greenhouse-grown *PdIQD10* RNAi downregulated lines relative to empty vector controls and a previously reported comparator *PdKOR* RNAi line with reduced growth phenotype (Figure 7A) (Kalluri et al., 2016) showed that *PdIQD10* lines may have increased growth relative to controls. For a deep dive into phenotypic characterization of *PdIQD10* RNAi plants, three biological replicates each of three independent transformation lines of *PdIQD10* and empty vector constructs was undertaken. These assessments showed that *PdIQD10* RNAi lines displayed greater plant height, stem diameter and stem density (Figures 7C–E) as compared to the empty vector control.

**FIGURE 8:**
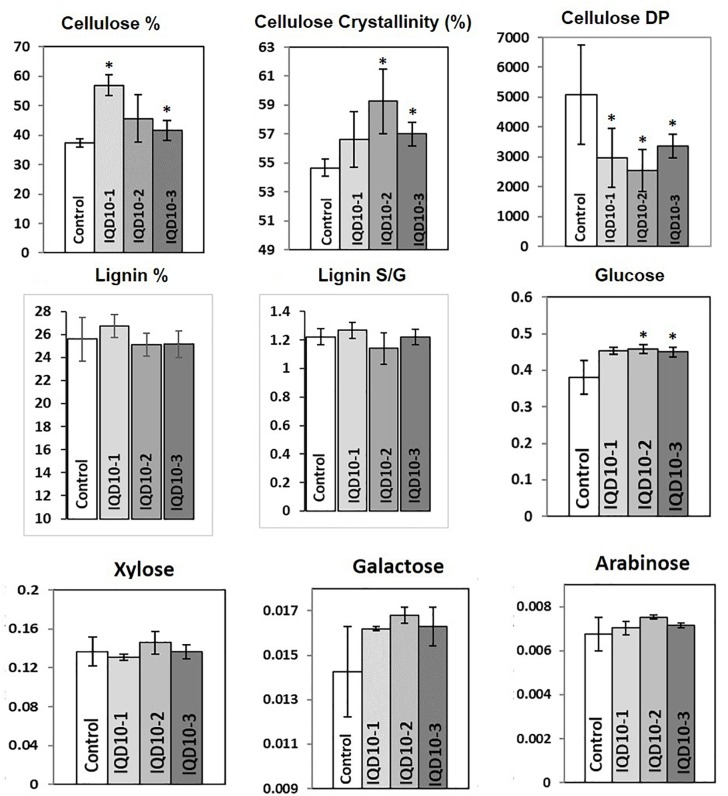
Cell wall characteristics of *PdIQD10* RNAi lines. Wet chemistry analysis of cellulose content, NMR analysis of cellulose crystallinity, GPC analysis of cellulose DP, MBMS analysis of lignin content and S/G ratio, and analysis of sugar composition of cell walls in control and three independent *PdIQD10* RNAi lines. Data represent means ± SD (*n* = 3–7). ^∗^Indicates statistically significant, *p* ≤ 0.05 based on Student’s *t*-tests.

Sugar composition analysis of cell wall showed higher glucose level but no significant consistent change in galactose, xylose, and arabinose content in independent RNAi lines relative the control (Figure 8). Altered glucose level in *PdIQD10* RNAi lines relative to control suggest the potential functional significance of PdIQD10 in wall cellulose and hemicellulose composition and secondary cell wall biosynthesis pathways. Wet chemistry-based quantification of cellulose content in dried stem samples showed a higher percentage of cellulose in the *PdIQD10* RNAi samples relative to control (Figure 8). NMR techniques showed that a higher cellulose crystallinity, while gel permeation chromatography analysis showed a lower degree of polymerization for cellulose in the RNAi samples (Figure 8). MBMS analysis of lignin content and S/G ratios suggests that the impact of *PdIQD10* RNAi on lignin content was not significant (Figure 8).

### PdIQD Interacting KLC Proteins Show Transactivator Function

**FIGURE 9:**
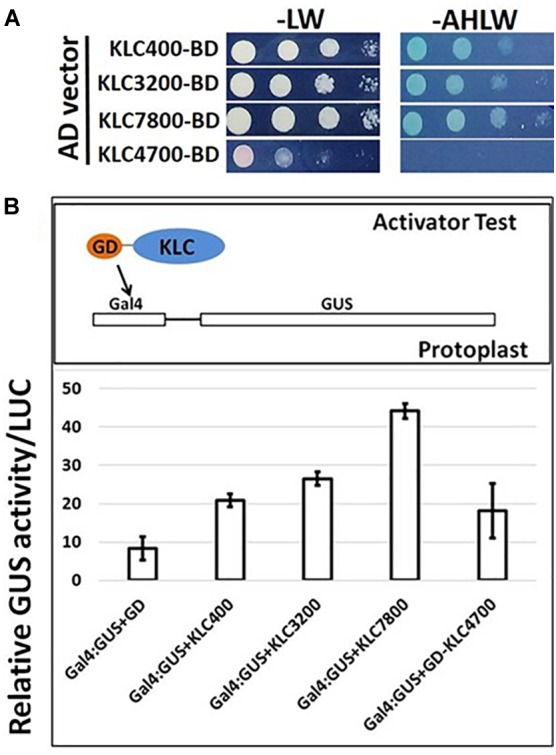
Transactivation analysis of KLC proteins. **(A)** Yeast two-hybrid assays with KLCs in BD vector co-transfected with empty AD vectors and grown on minimal media lacking adenine, histidine, leucine and tryptophan. **(B)** Protoplasts co-transfected with GD-fused KLCs along with *Gal4*:GUS reporter construct display GUS activity.

In addition to proposed cellular roles of KLC proteins in plants in signal and cargo transport along the microtubular network, a cellular functional role in transcriptional activation has also been reported in literature (Li et al., 2011, 2012). These studies reported that BC12/GDD1 (Gibberellin-Deficient Dwarf1), a rice Kinesin-Like Protein that is bound to microtubules in an ATP-dependent manner also binds to the promoter of ent-kaurene oxidase (KO2) – an enzyme in gibberellic acid (GA biosynthesis). Protoplast assays revealed that GDD1 has transcriptional activation activity and that T-DNA insertion line *gdd1* has reduced accumulation of GA. Our Yeast two-hybrid experiments designed to test such a transactivator ability for *Populus* KLCs showed that three out of four KLC protein isoforms were able to autoactivate the transcription of reporter genes *HIS3*, *ADE2* and *MEL1* without the interacting partner protein (Figure 9A). To rule out the possibility that this might be the result of targeted nuclear localization in yeast two-hybrid system and to test these results *in planta*, we made use of *Populus* protoplast transient system. The Gal4 DNA binding domain (GD) fused *KLCs* were co-transfected with *Gal4:GUS* reporter construct to test if KLCs induce GUS transcript activation. GD-KLCs were able to activate the transcription of *Gal4* promoter-fused GUS reporter suggesting the transcriptional activation functions of KLCs (Figure 9B). GD-KLC7800 transformation induced highest GUS activity indicating its relatively stronger transactivation ability relative to GD-KLC3200, GD-KLC400, and GD-KLC4700, displaying lower and differential transactivation strengths.

**FIGURE 10:**
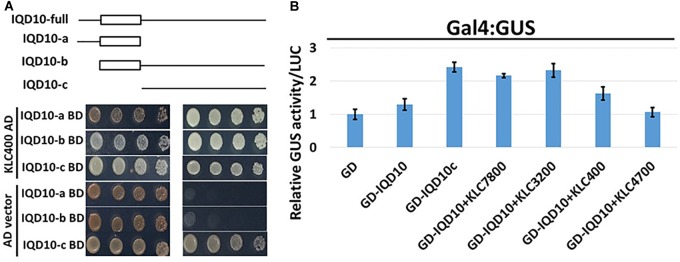
Domain split analysis of PdIQD10 using transactivation assay. **(A)** Yeast two-hybrid analysis of PdIQD10 domains with KLC400 indicates the possible interaction of KLC400 with IQD10a and IQD10b fragments. Also, the empty vector AD control transfection with split PdIQD10 domains in BD shows autoactivation indicating its transactivation functions. **(B)** Protoplasts co-transfected with *Gal4*:GUS reporter construct with versions of PdIQD10 in GD-fused constructs, in combination or not with KLCs, show increase in GUS activities indicating increased transactivation activity of PdIQD10 upon interaction with KLCs.

Yeast two-hybrid analysis to dissect the interacting domains of PdIQD10 with KLCs provided additional insights. PdIQD10 protein spanning from the initial start codon to the end of PdIQD10-domain is referred to here as IQD10a, from start of the PdIQD10-domain up till the stop codon as IQD10b and from the end of PdIQD10-domain up to the stop codon as IQD10c (Figure 10A). Yeast transformants carrying Binding Domain (BD) fused IQD10c was able to autoactivate the transcription of HIS3 reporter thus inducing Yeast growth on minimal media lacking Hisidine, Leucine and Tryptophan (Figure 10A) without the interacting partner KLC400. This observation indicated two mechanisms: (i) IQD10c might have transactivation functions and (ii) The N-terminal PdIQD10-domain fragment might be involved in suppressing the transactivation activity of IQD10c as the full-length BD fused PdIQD10 does not show autoactivation in Yeast (Figures 3A, 10 and Supplementary Figure S8). Based on these observations, transactivation properties of PdIQD10 full length and IQD10c proteins were tested with or without KLC protein co-transfection in *Populus* protoplasts.

The protoplasts transfected with empty GD vector and GD fused PdIQD10 displayed weak GUS activity to the same extent suggesting full length PdIQD10 has no transactivation activity (Figure 10B). However, transfection of GD-fused IQD10c induced GUS activity more than twice that of the GD control and full length PdIQD10 indicating GD-IQD10c may be able to activate the *Gal4* promoter. Furthermore, co-transfections with KLC7800 and KLC3200 showed induction of PdIQD10 transactivation activity to a similar level as IQD10c, which is higher relative to control (Figure 10B). These results suggest a potential activation effect of KLC proteins on PdIQD10.

## Discussion

The present study provides evidence in support of a functional role for a new calmodulin-binding protein member in the contexts of secondary cell wall biosynthesis based on expression and molecular biology studies, and in the context of biomass formation based on transgenic *Populus* plant characterization.

### PdIQD10 Is Preferentially Expressed in the Context of Secondary Cell Wall Formation and Is Potentially Regulated by Secondary Cell Wall Transcription Factors

The transcript accumulation of *PdIQD10* was significantly higher in tension-stressed, secondary wall-enriched, xylem tissue along with other known cell wall marker genes such as sucrose synthase (*SUSY*), cellulose synthases (*CESAs*), and *KOR* (*KORRIGAN*) (Figure 1A). The qRTPCR assay of *PdIQD10* gene expression showed highest levels in xylem tissue (>100-fold) relative to other tissue/organ libraries (Figure 1B).

Promoter binding assays support a functional context for *PdIQD10* in the secondary cell wall biosynthesis transcriptional regulatory network where the *Populus* homolog of the known master regulator SND1, PdWND1B, binds to *PdHB3* promoter and PdHB3 protein in turn binds to *PdIQD10* promoter.

### PdIQD10 Is Potentially a Component of Multi-Protein Signaling Complex

**FIGURE 11:**
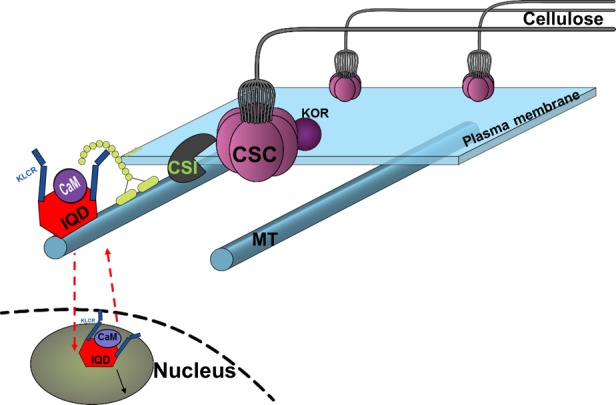
Conceptual model of proposed functional role of PdIQD10 in cell wall biosynthesis pathway at both its plasma membrane and nucleus (nuclear region) locations. The conceptual localization of PdIQD10 on microtubules (MT) is based on similar studies in *Arabidopsis* and not undertaken or evidenced in the present study of *Populus*.

The members of the IQD family are known for their interaction with the calmodulin and calmodulin-like proteins (Bahler and Rhoads, 2002; Abel et al., 2013; Bürstenbinder et al., 2013, 2017b). We have shown that the full-length PdIQD10 interacts weakly with CaM014 and CaM2599 out of 26 CaMs in yeast-two-hybrid assays. The PdIQD10-domain interacts with CaM247 and CaM351 but not CaM014 and CaM2599. In agreement with *Arabidopsis* IQD1 studies (Bürstenbinder et al., 2013), PdIQD10 was found to interact with KLC proteins (Figure 6). Subcellular localization experiments reveal that PdIQD10, CaM014 and CaM2599 localize to the plasma membrane and the nucleus (Figures 4A–D). These observations indicate the possible complex formation between PdIQD10, CaMs and the kinesin-light chain proteins, similar to the model proposed for *Arabidopsis* IQD1 protein complex (Bürstenbinder et al., 2013) (Figure 11).

The putative PdIQD10 interacting partners proteins selected from various databases (Table 3) either have known cell wall related functions or represent potential members in cell wall or stem development pathways. Yeast two hybrid assays showed weak or no interactions with selected co-expressing factors such as NEK5, NEK6, PtiCesA7-A, IRX10, GAUT12.1, SCPL14, and SCPL41(Supplementary Figure S8). The weak interactions may be due to the physiology of the yeast and the lack of the functional scaffolding protein for two proteins to interact or a measure of no functional competence for biological interaction between targeted proteins. CesA complexes (CSCs) are known to move in plasma membrane guided by the underlying cortical microtubular network and directionally deposit cellulose microfibrils in the cell wall (Gu et al., 2010; Endler and Persson, 2011). Studies from Lei et al. provided evidence that CSI interact with CesAs and regulate their function in a microtubule dependent or independent manner (Lei et al., 2013) as well as their turnover or recycling from plasma membrane (Lei et al., 2015). Golgi complex and vesicle trafficking network are integral to the cellulose biosynthesis process with CSCs known to assemble in the Golgi complex. More recently, a Golgi-localized protein, STELLO, has been identified as a key regulator of cellulose biosynthesis via its functional role in assembly of CSCs in Golgi and its trafficking to the plasma membrane (Zhang et al., 2016). These recent studies suggest there are additional signaling and regulatory factors involved in the coordinated recruitment of CSCs to microtubules, and their integration into and turnover in plasma membrane. Functional characterization of proteins co-expressing and/or interacting with factors implicated in cell wall pathways would lead to new insights. Based on the findings in the present study, we hypothesize that cell wall biosynthesis components or signals regulating the bioprocess may be carried as potential cargo in PdIQD10-KLC mediated directional transport along the microtubules (Bürstenbinder et al., 2013) (Figure 11).

### PdIQD10 Is a Potential New Signaling Factor in the Secondary Cell Wall and Stem Growth Bioprocesses

Our protein interaction studies support the role of predicted calmodulin-binding *PdIQD10* gene as a CaM-binding protein. CaMs and Ca^2+^-signaling encompasses a core signaling mechanism to mediate signal transduction from plasma membrane, cytosolic and nuclear compartments. Our subcellular localization assays show that PdIQD10 localizes both in the nucleus and plasma membrane as well as bind to other known xylem expressing, signaling complex proteins. Considering IQD’s basic cellular role in Ca^2+^/CaM signaling and the strong expression of this particular *IQD*, *PdIQD10*, in the context of cells undergoing secondary wall biosynthesis, along with phenotypic ramifications on secondary cell wall chemistry and biomass formation, we propose that PdIQD10 is a potential new signaling factor in the secondary cell wall and stem growth bioprocesses. Follow-on studies are needed to clarify the functional role as general or specific to aspects of cell wall formation.

The nuclear localization and transactivation ability of KLC proteins and their influence on the transactivation activity of PdIQD10 suggest that the complex of PdIQD10-KLCs may potentially regulate genes involved in secondary cell wall biosynthesis or stem developmental processes. Further studies are needed to clarify the additional protein composition of the complex and genes regulated by such a complex and their cascading mechanistic influence on *Populus* biomass properties.

RNAi lines interestingly displayed increased biomass formation as reflected by their increased plant height, stem diameter and stem density. RNAi plant stem samples also showed a changes in cellulose properties and wall sugar composition relative to control stems. The observation of increased stem growth and cellulose content via knockdown of *PdIQD10* is interesting, which leads to the new hypothesis that this gene, in part, partakes in mediating the tight feedback-loop in regulation of cellulose content and biosynthesis in secondary walls in *Populus* stems. The known co-expression of *PdIQD10* with other secondary cell wall polysaccharide pathway genes such as *PdIRX10*, beta-1,4-xylosyltransferease, a homolog of *AtIRX10* implicated in hemicellulose biosynthesis, combined with the observation that *PdIQD10* RNAi lines have altered wall glucose levels relative to control, suggest a potential role of *PdIQD10* gene in secondary cell wall (cellulose and hemicellulose) biosynthesis pathways. Our yeast-two-hybrid assays with PdIQD10 and selected proteins identified to be co-expressing from public databases showed weak or no interactions (Supplementary Figure S8). Given the frequency of false positives and false negatives in yeast-two hybrid assays in general, observations here will need alternate lines of evidence such as *in vivo* protein-protein interactions, protein pull-down assays or computational interactome predictions.

## Conclusion

The present study provides evidence in support of a new functional context for an IQD family member in secondary cell wall biosynthesis and biomass formation. Specifically, the study shows that *PdIQD10* gene codes for a calmodulin-binding protein, expresses under tissue contexts of higher secondary cell wall formation, potentially regulated by secondary cell wall transcription factor, has subcellular localization contexts of plasma membrane and nucleus, and forms protein complexes with CaM and KLC proteins. Stable knockdown of gene expression in transgenic *PdIQD10* RNAi *Populus* plants impacted the properties of resultant stem biomass. *PdIQD10* RNAi plants displayed enhanced growth, accompanied by quantitatively modest, yet significant, concomitant changes in cellulose content and crystallinity and wall sugar composition relative to the control.

In the future, quantitative analysis of cell wall pathway and interactors proteins in *PdIQD10* RNAi and control lines and pulldown assays with tagged-*IQD* overexpression lines will aid in clarifying PdIQD10’s involvement in signaling and response for biosynthesis of a specific wall component, cellulose, wall remodeling and integrity sensing, and/or stem developmental programs. The significant phenotypic effect on cellulose properties observed here will require clarification of a direct impact on cellulose biosynthesis pathway by imaging anomalies in movement or turnover of GFP-tagged CesAs in *IQD10*-modified plants. Experimental studies designed to probe the binding potential of PdIQD10 to microtubule proteins in the presence or absence of calcium ions or calcium chelators would further inform its molecular activity as a signaling protein. While the present study is a first characterization of a single isoform from the large IQD protein family in *Populus*, the full functional repertoire of the large *IQD* gene family in *Populus* is still unknown. Last but not the least, garnering functional genomics evidence for additional genes of unknown function strongly co-expressing with xylem or secondary cell wall development marker genes will greatly expand the critical knowledge base needed to understand and optimize plant cell wall properties.

## Author’s Note

This manuscript has been authored by UT-Battelle, LLC, under contract DE-AC05-00OR22725 with the US Department of Energy (DOE). The US government retains and the publisher, by accepting the article for publication, acknowledges that the US government retains a non-exclusive, paid-up, irrevocable, worldwide license to publish or reproduce the published form of this manuscript, or allow others to do so, for US government purposes. DOE will provide public access to these results of federally sponsored research in accordance with the DOE Public Access Plan (http://energy.gov/downloads/doe-public-access-plan).

## Author Contributions

RB designed and conducted experiments to understand molecular function including protein-interaction assays and wrote manuscript. RP designed and conducted transgenic plant phenotyping, expression analysis and wrote manuscript. SJ and LG carried out plant growth and phenotyping. XY designed the RNAi construct, H-BG conducted MD simulations. KW, CC, and WR generated transgenic plants in tissue culture and stool beds. KY and MR conducted wall chemistry assays. RS, SD, and MD carried out MBMS and biomass sugar assays. GB and AR conducted NMR and GPC analysis of cellulose. GT and UK conceived the study and wrote the manuscript.

## Conflict of Interest Statement

The authors declare that the research was conducted in the absence of any commercial or financial relationships that could be construed as a potential conflict of interest.

## Footnotes

1 http://string-db.org/

2 http://atted.jp/

3 https://phytozome.jgi.doe.gov/pz/portal.html#

4 https://phytozome.jgi.doe.gov

5 http://version10.string-db.org/cgi/input.pl

6 http://atted.jp/

## Supplementary Material

Figure S1 Construct design of the *PdIQD10* RNAi construct.

Figure S2 Expression analysis of *PdIQD10* in LCM-derived stem tissue and xylem cell types of *Populus* using database published by Shi et al. (2017).

Figure S3 Multiple sequence alignment of CaM014 and CaM2599 protein sequences.

Figure S4 Multiple sequence alignment of CaM247, CaM014, and CaM2599 protein sequences showing the shorter N-terminal region of CaM247.

Figure S5 Phylogenetic tree of calmodulin/calmodulin-like (*CaM*) gene family members of *Populus* and *Arabidopsis*. The tree was generated using Maximum Likelihood algorithm in MEGA7.0.25 with 1000 bootstrap replicates and represents 36 *Populus* and 25 *Arabidopsis* calmodulin/calmodulin-like protein sequences.

Figure S6 Subcellular localization of CaM247 and CaM014 in *Populus* protoplasts. To increase accessibility of these subcellular localization images, the yellow color channel was converted to magenta uniformly across all images in the CMYK color spectrum. The original RGB color scheme images are provided in Supplementary Figure S10. The color scheme is as follows: GFP/RFP: blue/cyan; chlorophyll, FM64 and mCherrry: red/orange.

Figure S7 Domain Architecture of the four *Populus* KLC proteins.

Figure S8 Yeast two-hybrid interaction analysis of PdIQD10 with proteins from co-expressing genes. Yeast two-hybrid interaction analysis of PdIQD10 with putative interacting proteins chosen from the database analysis results. Bar graph on the right represents the average β-galactosidase activities assayed from three independent yeast colonies to determine the strength of the interactions.

Figure S9 Expression analysis of *PdIQD10* gene in various *Populus* tissues. This figure is the original version of Fig1b showing the continuous Y-axis scale.

Figure S10 Original RGB subcellular localization images used to generate accessible images in Figure 4 and Supplementary Figure S6.

## Abbreviations

CaMs: calmodulins
CBLs: calcineurin B-like proteins
cDNA: complementary DNA
CDPK: CaP^2+^P-dependent protein kinases
CesA: cellulose synthase
Chi: chitinase-like protein
CHUP: chloroplast unusual positioning
CIPKs: CBL-interacting protein kinases
CMLs: Calmodulin-like proteins
CSC: cellulose synthase complex
CSI1: cellulose synthase-interactive protein 1
CTAB: Cetyl trimethylammonium bromide
DICE: defect in cell elongation
DOE: Department of Energy
DUF: Domain of Unknown Function
GAUT: Galacturonosyltransferase
GUS: β-glucuronidase
HD-ZIP III: class III homeodomain-leucine zipper protein
HYP: hypothetical protein with unknown function
IQD: IQ-domain containing protein
IRX10: beta-1,4-xylosyltransferease (irregular xylem phenotype)
KLCR or KLC: kinesin light chain-related protein
KOR: Korrigan
Lac: laccase
MAP: Microtubule-associated protein
MEGA: Molecular Evolutionary Genetics Analysis
NEK: NIMA-related kinases
NIMA: Never in mitosis A
ONPG: ortho-nitrophenyl-β-galactoside
RIC4: Rac-interactive binding
SCPL: Serine carboxypeptidase
SND: secondary wall-associated NAC domain protein
UC: plastocyanin-like domain (PCLD)-containing protein (UC-like)
UGPase: UDP-glucose pyrophosphorylase
UTR: Un-translated Regions
WND: wood-associated NAC domain transcription factor
