# Supplementary figures and images for: A New Calmodulin-Binding Protein Expresses in the Context of Secondary Cell Wall Biosynthesis and Impacts Biomass Properties in Populus
Source: Front Plant Sci. 2018 Dec 5;9:1669. doi: 10.3389/fpls.2018.01669 (PMC6290091; doi:10.3389/fpls.2018.01669)

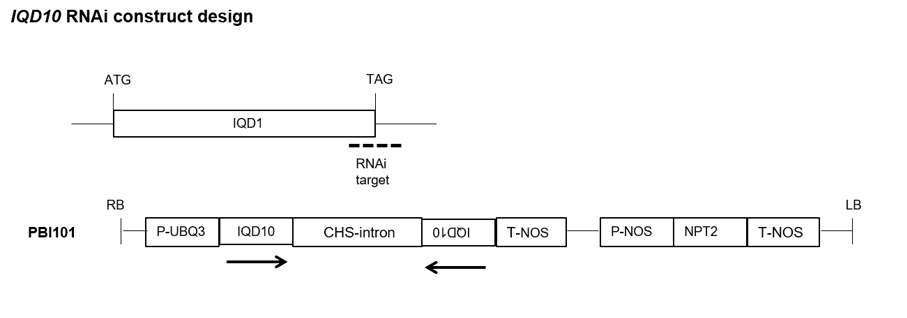

Supplement: Figure S1 — Construct design of the PdIQD10 RNAi construct. [file Image_1.TIF]

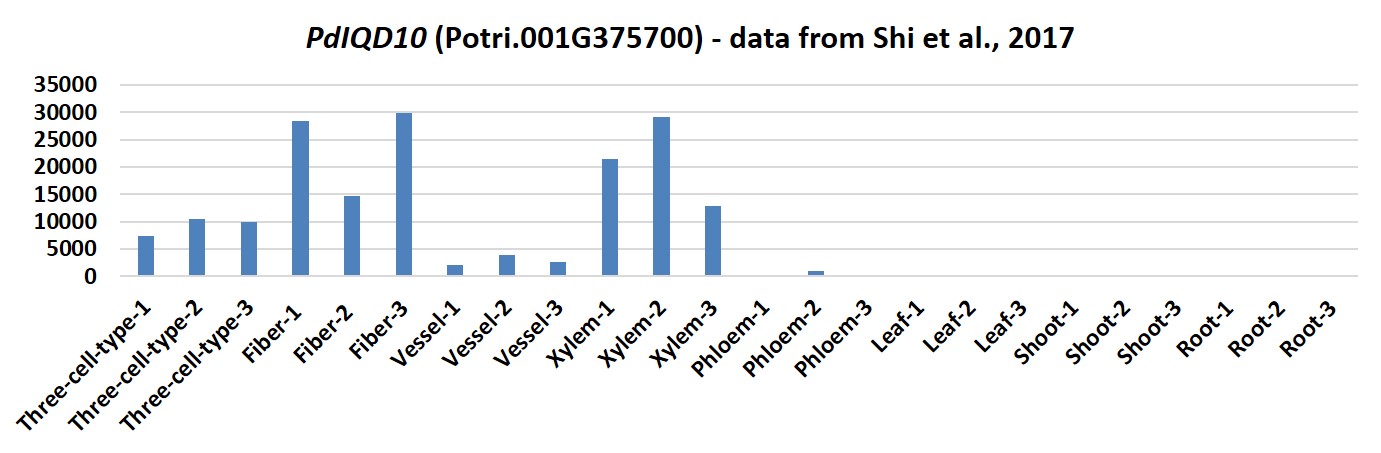

Supplement: Figure S2 — Expression analysis of PdIQD10 in LCM-derived stem tissue and xylem cell types of Populus using database published by Shi et al. (2017). [file Image_2.JPEG]

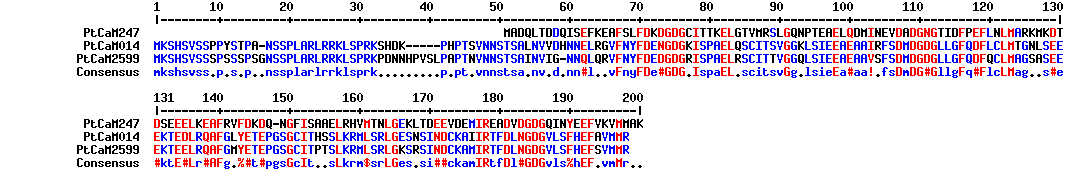

Supplement: Figure S4 — Multiple sequence alignment of CaM247, CaM014, and CaM2599 protein sequences showing the shorter N-terminal region of CaM247. [file Image_4.TIF]

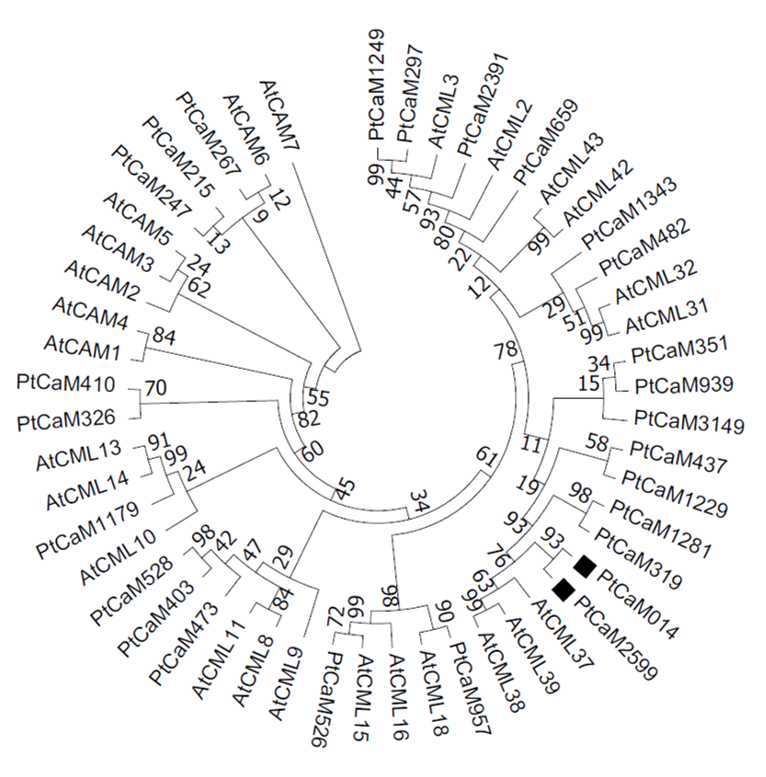

Supplement: Figure S5 — Phylogenetic tree of calmodulin/calmodulin-like (CaM) gene family members of Populus and Arabidopsis. The tree was generated using Maximum Likelihood algorithm in MEGA7.0.25 with 1000 bootstrap replicates and represents 36 Populus and 25 Arabidopsis calmodulin/calmodulin-like protein sequences. [file Image_5.TIF]

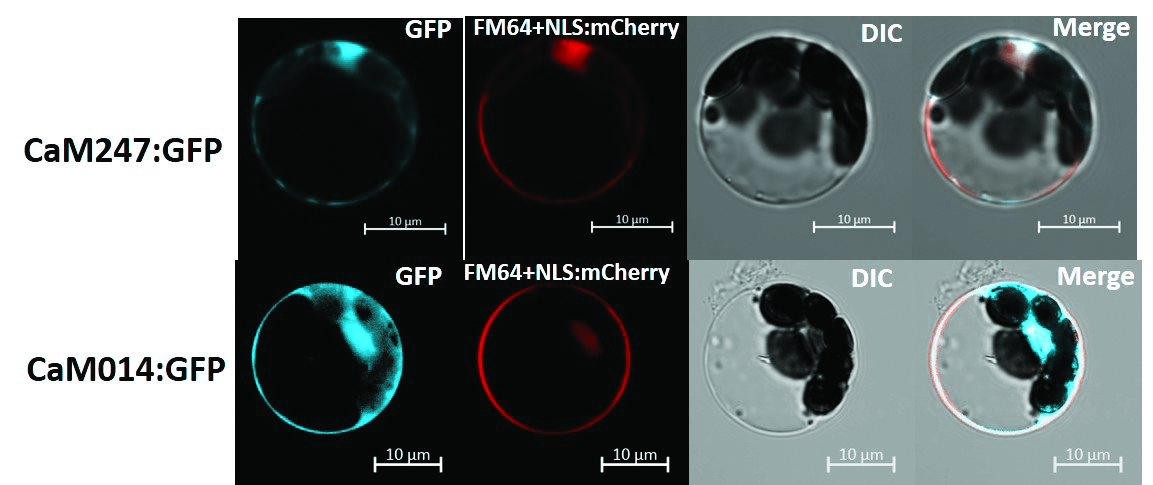

Supplement: Figure S6 — Subcellular localization of CaM247 and CaM014 in Populus protoplasts. To increase accessibility of these subcellular localization images, the yellow color channel was converted to magenta uniformly across all images in the CMYK color spectrum. The original RGB color scheme images are provided in Supplementary Figure S10. The color scheme is as follows: GFP/RFP: blue/cyan; chlorophyll, FM64 and mCherrry: red/orange. [file Image_6.JPEG]

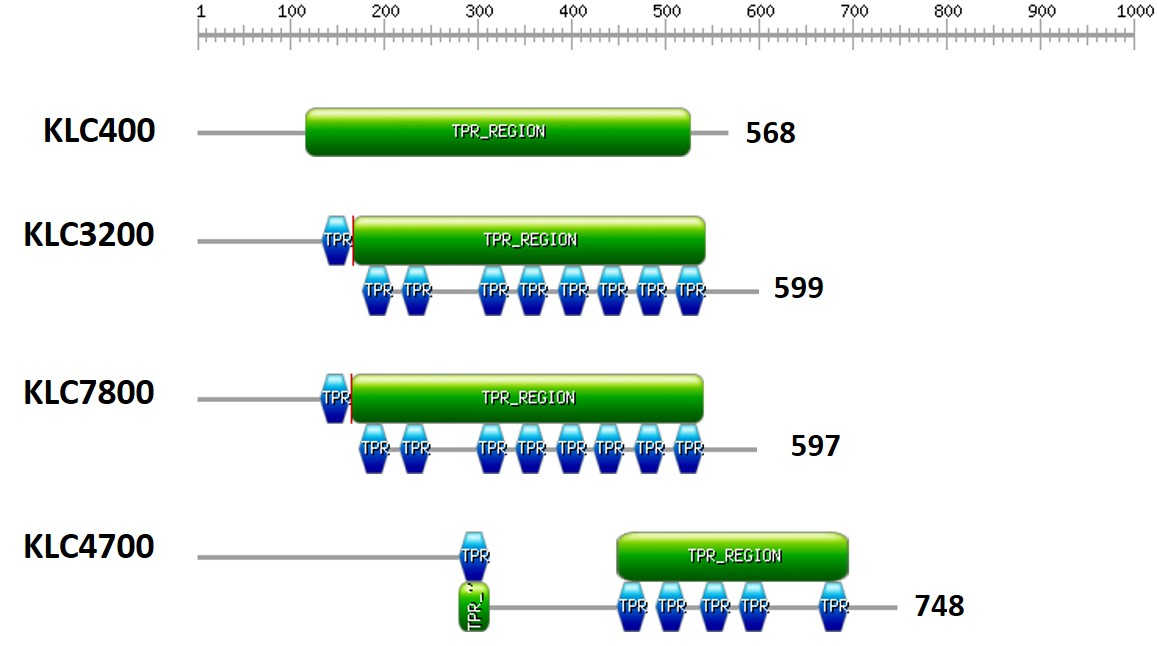

Supplement: Figure S7 — Domain Architecture of the four Populus KLC proteins. [file Image_7.TIF]

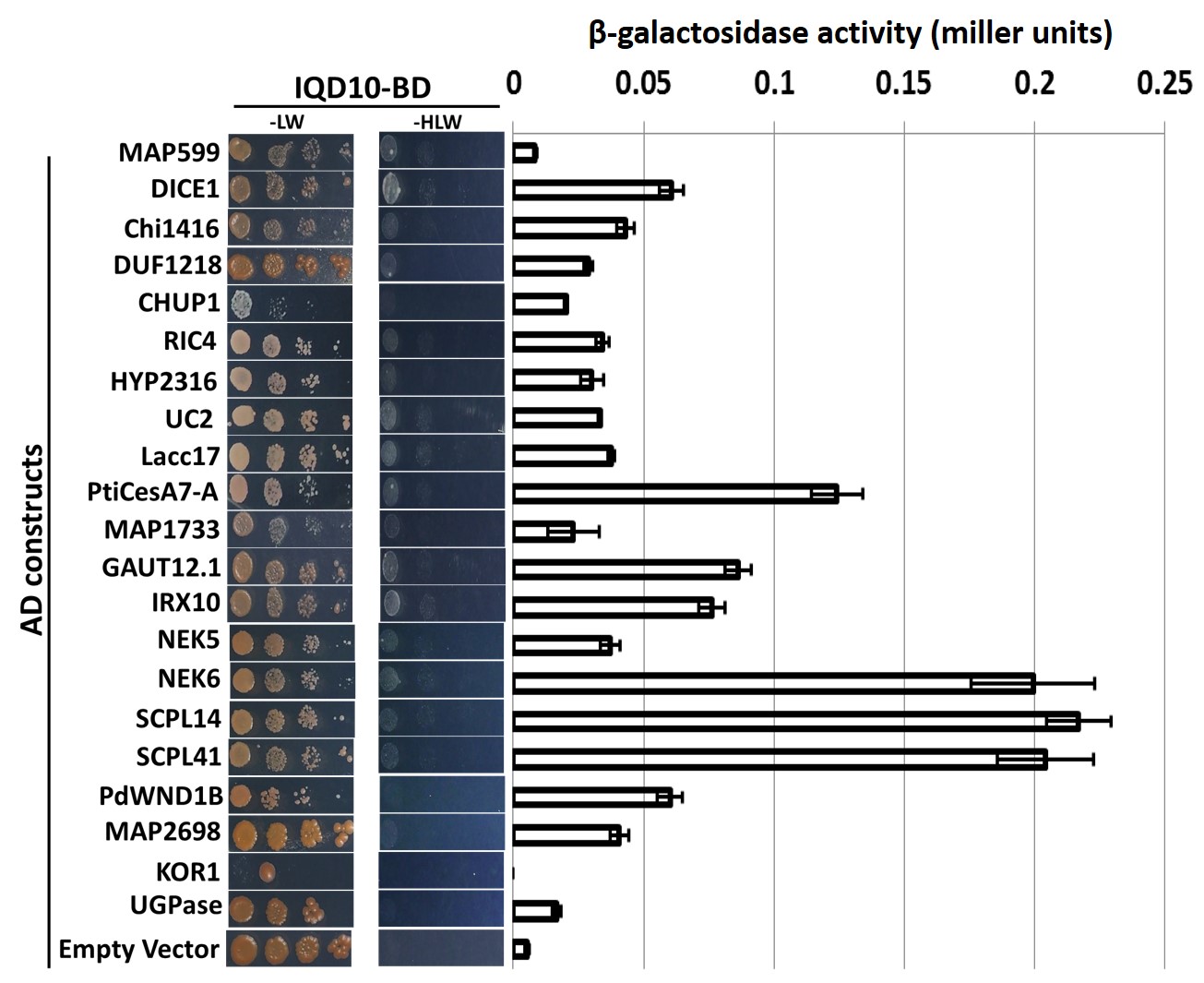

Supplement: Figure S8 — Yeast two-hybrid interaction analysis of PdIQD10 with proteins from co-expressing genes. Yeast two-hybrid interaction analysis of PdIQD10 with putative interacting proteins chosen from the database analysis results. Bar graph on the right represents the average β-galactosidase activities assayed from three independent yeast colonies to determine the strength of the interactions. [file Image_8.JPEG]

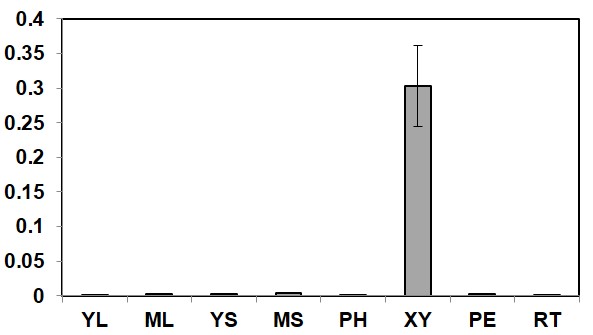

Supplement: Figure S9 — Expression analysis of PdIQD10 gene in various Populus tissues. This figure is the original version of Fig1b showing the continuous Y-axis scale. [file Image_9.JPEG]
