# Supplementary material for: A New Calmodulin-Binding Protein Expresses in the Context of Secondary Cell Wall Biosynthesis and Impacts Biomass Properties in Populus
Source: Front Plant Sci. 2018 Dec 5;9:1669. doi: 10.3389/fpls.2018.01669 (PMC6290091; doi:10.3389/fpls.2018.01669)
Supplement: Figure S10 — Original RGB subcellular localization images used to generate accessible images in Figure 4 and Supplementary Figure S6. [file Image_10.pdf]

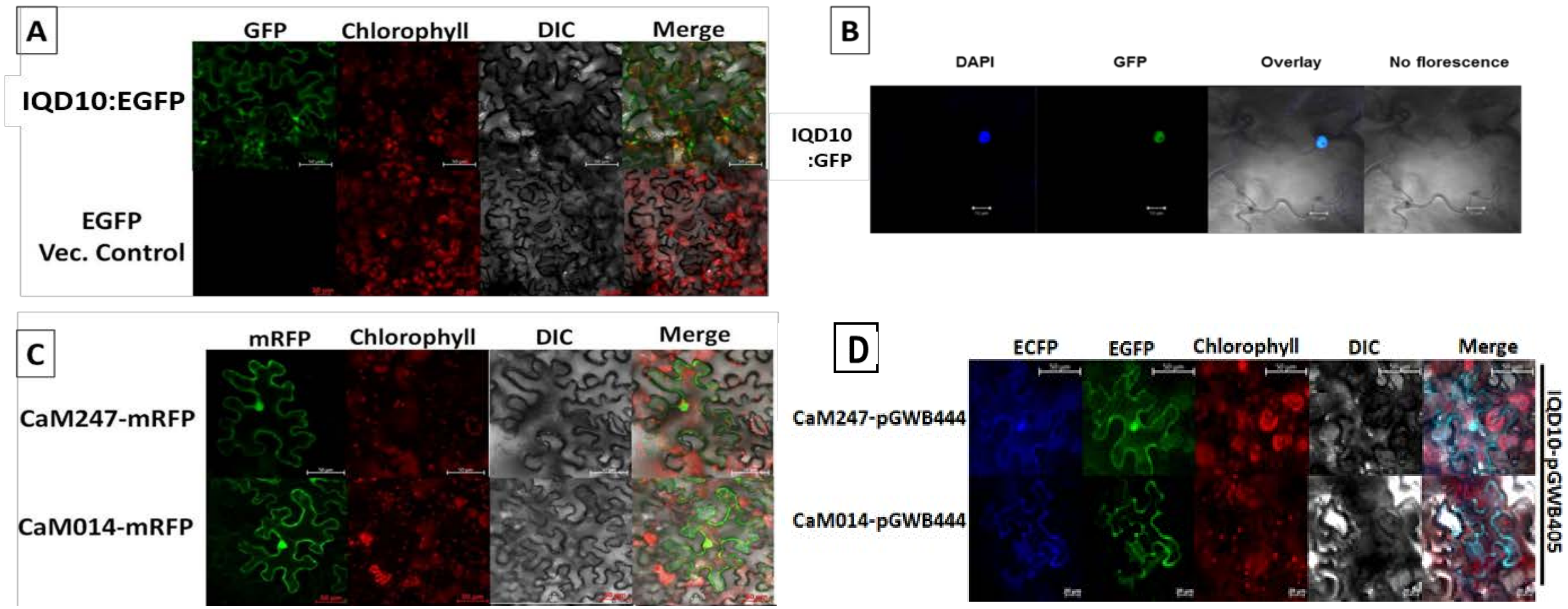

Supplemental Fig. 10. Original RGB subcellular localization image used to generate the accessible image in Fig. 4.

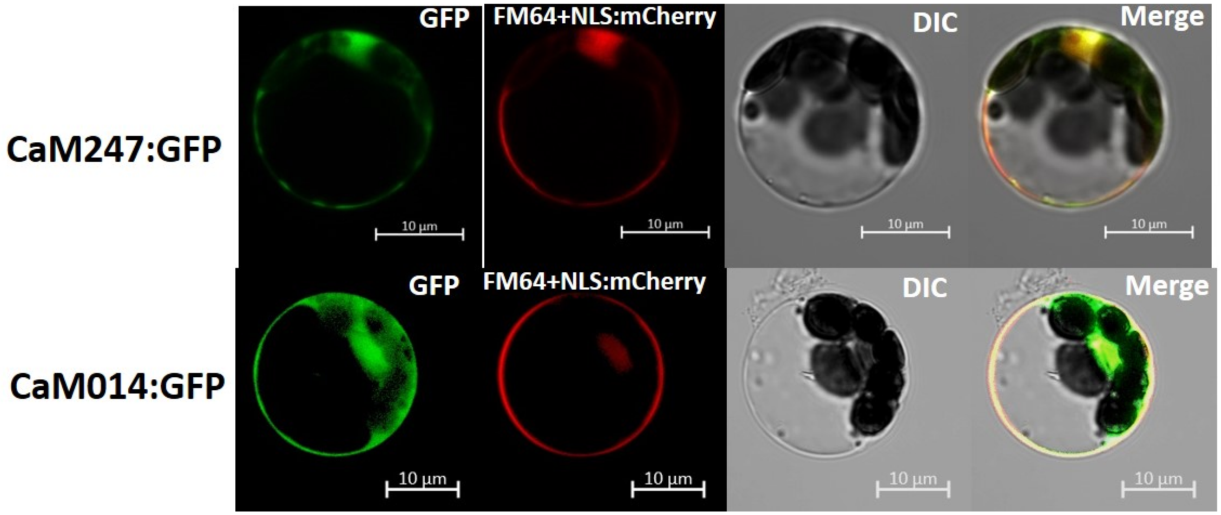

Supplemental Fig. 10. Original RGB subcellular localization image used to generate the accessible image in Supplemental Fig. 6.
